# Proceedings of the 2017 WAO Symposium on Hot Topics in Allergy: Pediatric & Regulatory Aspects: Rome, Italy/Vatican City. 27-29 April 2017

**DOI:** 10.1186/s40413-017-0170-3

**Published:** 2017-11-16

**Authors:** Giovanni Traina, Rocco Luigi Valluzzi, Vincenzo Fierro, Carla Riccardi, Maria Cristina Artesani, Andrea De Vuono, Alessandro Fiocchi, Alberto G. Martelli, Luis Alberto Ríos, Christian R. Alcocer, Elsy Navarrete, Blanca Estela Del Rio Navarro, Victor Gonzalez, Berenice Velasco, Herberth J. Perez Aviles, Roberto Jose Fernandez, F. Cesar Pozo, Abdal Jabbar Farhan, Hasan Arshad, Ahmed Hussain, Olena Sharikadze, Olena Okhotnikova, Javier Alcover, Diego Rodriguez, Fernando Pineda, Ilan Dalal, Jenny Weinbrand-Goichberg, Shira Benor, Menachem Rottem, Shmuel Kivity, Sakura Sato, Noriyuki Yanagida, Motohiro Ebisawa, Tetiana Umanets, Fernando Pineda, Youriy Antipkin, Vladyslava Barzylovich, Volodymyr Lapshyn, Tetiana Umanets, Mykola Umanets, Sergey Yuriev, Fernando Pineda, Diego Rodriguez, Javier Alcover, Suzan Bekir, Tobias Pincock, Alberto Vieira Hernandez, Arnaldo Capriles Hulett, Mario Sánchez Borges, Fabiola Fabiano, Carlos Albarran, Rohit Goyal, Shilpa Gupta, Garg Gaurav, Allan T. Luskin, Noelle M. Griffin, Amy Wagelie-Steffen, Benjamin L. Trzaskoma, Susan L. Limb, William W. Busse, Robert S. Zeiger, Erika Gonzalez-Reyes, Thomas B. Casale, Bradley E. Chipps, Chizuko Sugizaki, Fumiko Goto, Sakura Sato, Noriyuki Yanagida, Motohiro Ebisawa, Akiko Yamaide, Kanako Mitsunaga, Minako Tomiita, Akira Hoshioka, Naoki Shimojo, Liviu L. Pop, Ioana-Mihaela Ciucǎ, Liviu Tǎmaş, Marilena Lazarescu, Corina Pienar, Fumiya Yamaide, Bahrul Fikri, Hironori Sato, Naoki Shimojo, Naoko Okishima, Miyabi Kobayashi, Mizuki Takai, Kotarou Nishigata, Ryou Yoda, Yu-ta Oana, Chifu Kajiwara, Moe Shimodaira, Tomoka Suzuki, Hiromi Iizawa, Koji Kamijo, Bijoya Karmakar, Swati Gupta Bhattacharya, Simona Blohlávková, Eliška Kopelentová, Petr Víšek, Jakub Štádler, Ivana Šetinová, Jana Novobílská, Krisa Lundelin, Seppo Salminen, Erika Isolauri, Tracy Pitt, Tamar Flanders, Marcos Peñalver, Patricia Martínez, Magdalena Lluch, Alfonso Malet, Young-Hee Nam, Hyun Jung Jin, Soo-Keol Lee, Prapasri Kulalert, Paskorn Sritipsukho, Jayanton Pathumanond, Krasimira Baynova, Marina Labella, Teresa De Aramburu, Manuel Prados, Leena Haanpää, Jasmin Aarnio, Merja Nermes, Piia af Ursin, Anne Kaljonen, Erika Isolauri, Nandana Bala, Ketaki Bhagwat, James Hindley, Martin Chapman, Sivasankar Baalasubramanian, Lilijana Besednjak-Kocijančič, Koyel SenGupta, Swati Gupta Bhattacharya, Bradley E. Chipps, Evgeniya Antonova, Amanda M. Kong, Ahmar Iqbal, W. Gerald Teague, Bradley E. Chipps, Evgeniya Antonova, Benjamin Trzaskoma, Benjamin Ortiz, Brandee Paknis, Amar Iqbal, Karin Rosen, Stanley Szefler, Afaf Alblooshi, Suleiman Al-Hammadi, Arantza Vega, Raquel Gutiérrez-Rivas, Ana Maria Alonso, Juan Maria Beitia, Maria Belén Mateo, Remedios Cárdenas, Juan Jesús García-Domínguez, Raquel Pitchon dos Reis, Cristina Gonçalves Alvim, Claudia Andrade, Adriana Reis, Henrique Ribeiro, Carmen Panaitescu Bunu, Laura Marusciac, Sorin Paralescu, Paul Tamas, Carmen Panitescu Bunu, Laura Marusciac, Sorin Paralescu, Paul Tamas, Enrique Martí Guadaño, Carolina Escobar Bolaños, Natalia Martí José, Pol Pau Casanovas, Gemma Biarnés Rib, Mariana Castells, Talía de Vicente Jiménez, Maurizio Mennini, Carla Riccardi, Papola De Angelis, Francesca Rea, Monica Malamisura, Renato Tambucci, Alessandro Fiocchi, Luigi Dall’Oglio, Maurizio Mennini, Federica Del Chierico, Tania Napolitano, Silvia Reddel, Pamela Vernocchi, Angelo D’Ambrosio, Lorenza Putignani, Maria Cristina Artesani, Lamia Dahdah, Vincenzo Fierro, Claudia Banzato, Luís Angel Echeverría Zudaire, Ana María Plaza, Montserrat Bosque García, Marcel Íbero, Oscar Mazzina, Vincenzo Fierro, Valeria Marzano, Carla Riccardi, Oscar Mazzina, Lamia Dahdah, Maurizio Mennini, Maria Cristina Artesani, Oscar Mazzina, Valeria Pecora, Pierluigi Koch, Rocco Luigi Valluzzi, Vincenzo Fierro, Alessandro Fiocchi, Valentina Pecora, Diletta Valentini, Maurizio Mennini, Lamia Dahdah, Oscar Mazzina, Francesca Santamaria, Rocco Valluzzi, Anwesha Mukherjee, Amit Kandhare, Subhash Bodhankar

**Affiliations:** 1 U.O.C Pediatria, Ospedale G. Salvini, Garbagnate Milanese, Milan, Italy; 2 Division of Allergy, University Department of Pediatrics, Pediatric Hospital Bambino Gesù, Rome, Italy; 3 Ufficio Statistica, Ospedale G. Salvini, Garbagnate Milanese, Milan, Italy; 4 Pediatric Allergy and Clinical Immunology, Hospital Infantil de México Federico Gómez, Mexico City, Mexico; 5 Pediatric and Neonatology Department, New Mowasat Hospital, Salmiya, Kuwait; 6 Faculty of Medicine, Southampton University, Southampton, UK; 7 Amiri Hospital, Kuwait City, Kuwait; 8 Shupyk National Medical Academy of Postgraduate Education, Kyiv, Ukraine; 9 Diater Laboratories, Madrid, Spain; 10 Pediatric Allergy Unit, E. Wolfson Medical Center, Pediatric Department, E. Wolfson Medical Center, Sackler faculty of Medicine, Tel Aviv University, Tel Aviv, Israel; 11 Pediatric Department, E. Wolfson Medical Center, Tel Aviv, Israel; 12 Allergy and Clinical Immunology Unit, Tel Aviv Sourasky Medical Center, Sackler Faculty of Medicine, Tel Aviv University, Tel Aviv, Israel; 13 Allergy Asthma and Immunology Unit, Emek Medical Center, Afula, Israel; 14 Department of Allergy, Clinical Research Center for Allergy and Rheumatology, Sagamihara National Hospital, Kanagawa, Japan; 15 Department of Pediatrics, Sagamihara National Hospital, Kanagawa, Japan; 16 Institute of Pediatrics, Obstetrics and Gynecology, Kyiv, Ukraine; 17 Diater Laboratories, Madrid, Spain; 18 Institute of Pediatrics, Obstetrics and Gynecology, Kyiv, Ukraine; 19 Heart Institute, Kyiv, Ukraine; 20 Ukrainian School of Molecular Allergy and Immunology, Kyiv, Ukraine; 21 Diater Laboratories, Madrid, Spain; 22 Collective.care Allergy Clinic, Sydney, Australia; 23 Australian Allergy Centre, Sydney, Australia; 24 Hospital de Emergencias de Naiguata, Naiguata, Venezuela; 25 Departamento de Alergologia, Hospital de San Juan De Dios, Caracas, Venezuela; 26 Departamento de Pediatria, Centro Medico Docente La Trinidad, Caracas, Venezuela; 27 Laboratorio Torre Caracas, Caracas, Venezuela; 28 Max Super Speciality Hospital, Bathinda, India; 29 Batra Hospital, New Delhi, India; 30 Deen Dayal Upadhyay Hospital, New Delhi, India; 31 HealthyAirways, Madison, WI USA; 32 Genentech, Inc., South San Francisco, CA USA; 33 Allergy Associates of Tucson, P.C., Tucson, AZ USA; 34 University of Wisconsin School of Medicine and Public Health, Madison, WI USA; 35 Deptartment of Allergy and Research and Evaluation, Kaiser Permanente Southern California, San Diego, CA USA; 36 The Children’s Hospital of San Antonio, Baylor College of Medicine, San Antonio, TX USA; 37 Division of Allergy and Immunology, University of South Florida, Tampa, FL USA; 38 Capital Allergy and Respiratory Disease Center, Sacramento, CA USA; 39 Clinical Research Center, Sagamihara National Hospital, Sagamihara, Japan; 40 Department of Allergy and Rheumatology, Chiba Children’s Hospital, Chiba, Japan; 41 Department of Pediatrics, Chiba University Graduate School of Medicine, Chiba, Japan; 42 Department of Pediatrics II, Victor Babeş University of Medicine and Pharmacy Timisora, Timisoara, Romania; 43 Department of Biochemistry, Victor Babeş University of Medicine and Pharmacy Timisora, Timisoara, Romania; 44 National Cystic Fibrosis Center, Timisoara, Romania; 45 Department of Pediatrics, Graduate School of Medicine, Chiba University, Chiba, Japan; 46 Department of Health and Nutritional Science, Matsumoto University, Matsumoto, Japan; 47 Health Support Station, Matsumoto University, Matsumoto, Japan; 48 JDA-DAT, Matsumoto, Japan; 49 Senior Research Fellow, Bose Institute, Kolkata, India; 50 Senior Professor, Bose Institute, Kolkata, India; 51 Immuno-flow, s.r.o., Prague, Czech Republic; 52 Medical Faculty, Charles University, Pilsen, Czech Republic; 53 Allergology Department, Kolín Hospital, Kolín, Czech Republic; 54 Allergology and Immunology Department, Faculty Hospital Motol, Prague, Czech Republic; 55 Allergology and Immunology Department, Litomyšl, Czech Republic; 56 Immunia, s.r.o., Prague, Czech Republic; 57 Alergollogy Novobílská, Vratimov, Czech Republic; 58 Department of Paediatrics, Turku University Hospital, Turku, Finland; 59 Functional Foods Forum, University of Turku, Turku, Finland; 60 Department of Paediatrics, Humber River Hospital, Toronto, ON Canada; 61 Deptartment of Paediatrics, Queen’s University, Kingston, ON Canada; 62 Midtown Paediatrics, Toronto, ON Canada; 63 Probelte Pharma, Murcia, Spain; 64 Allergy and Clinical Immunology Service, Hospital de Nens de Barcelona, Barcelona, Spain; 65 Department of Internal Medicine, College of Medicine, Dong-A University, Busan, Korea; 66 Department of Internal Medicine, Yeungnam University Medical School, Daegu, Korea; 67 Division of Clinical Epidemiology, and Division of Allergy and Immunology, Deptartment of Pediatrics, Faculty of Medicine, Thammasat University (Rungsit Campus), Pathum Thani, Thailand; 68 Thammasat University Center of Excellence of Asthma, Allergy and Immunology, Thammasat University Hospital, Pathum Thani, Thailand; 69 Division of Allergy and Immunology, Department of Pediatrics, Faculty of Medicine, Thammasat University (Rungsit Campus), Pathum Thani, Thailand; 70 Center of Excellence in Applied Epidemiology, Thammasat University (Rungsit campus), Pathum Thani, Thailand; 71 Division of Clinical Epidemiology, Faculty of Medicine, Thammasat University (Rungsit Campus), Pathum Thani, Thailand; 72 Allergy Unit, University Hospital Virgen del Rocío, Seville, Spain; 73 Child and Youth Research Institute, Department of Clinical Medicine, University of Turku, Turku, Finland; 74 Department of Pediatrics and Adolescent Medicine, Turku University Hospital, Turku, Finland; 75 Faculty of Medicine, Department of Clinical Medicine, University of Turku, Turku, Finland; 76 Indoor Biotechnologies India Pvt Ltd, Bangalore, India; 77 Rainbow Children’s Hospital, Bangalore, India; 78 Indoor Biotechnologies Ltd, Cardiff, Wales UK; 79 Indoor Biotechnologies Inc, Charlottesville, VA USA; 80 Zdravstveni dom Nova Gorica, and Primary Pediatric Centre, Nova Gorica, Slovenia; 81 Division of Plant Biology, Bose Institute, Kolkata, India; 82 Capital Allergy and Respiratory Disease Center, Sacramento, CA USA; 83 Genentech, Inc., South San Francisco, CA USA; 84 Truven Health Analytics, an IBM Company, Cambridge, MA USA; 85 Child Health Research Center, University of Virginia School of Medicine, Charlottesville, VA USA; 86 Capital Allergy and Respiratory Disease Center, Sacramento, CA USA; 87 Genentech, Inc., South San Francisco, CA USA; 88 Novartis Pharmaceuticals Corporation, East Hanover, NJ USA; 89 Pediatric Asthma Research Program, Children’s Hospital and University of Colorado School of Medicine, Colorado, Aurora, CO USA; 90 Deptartment of Pediatrics, UAE University, Al-Ain, United Arab Emirates; 91 Allergy Service, Hospital Universitario de Guadalajara, Guadalajara, Spain; 92 Electronic Department, Polytechnic School, University of Alcalá, Madrid, Spain; 93 Department of Pediatrics, Federal University of Minas Gerais, Belo Horizonte, Brazil; 94 Allergy Department, Mater Dei Hospital, Belo Horizonte, Brazil; 95 Victor Babes University of Medicine and Pharmacy, Timisoara, Romania; 96 OncoGen Research Center, Emergency Clinical County Hospital, Timisoara, Romania; 97 Victor Babes University of Medicine and Pharmacy, Timisoara, Romania; 98 OncoGen Research Center, Emergency Clinical County Hospital, Timisoara, Romania; 99 Allergy Unit, Institut Català d’Oncològia, Barcelona, Spain; 100 UDAM Allergy Unit, Barcelona, Spain; 101 Desensitization Unit, Brigham and Women’s Hospital, Boston, MA USA; 102 Pediatrics & Allergy Department, Hospital Central de la Defensa Gómez-Ulla, Madrid, Spain; 103 Division of Allergy, University Department of Pediatrics, Pediatric Hospital Bambino Gesù, Rome, Vatican City Italy; 104 Digestive Surgery and Endoscopy Unit, Pediatric Hospital Bambino Gesù, Rome, Vatican City Italy; 105 Division of Allergy, University Department of Pediatrics, Pediatric Hospital Bambino Gesù, Rome, Vatican City Italy; 106 Human Microbiome Unit, Bambino Gesù Children Hospital, Rome, Italy; 107 Division of Allergy, University Department of Pediatrics, Pediatric Hospital Bambino Gesù, Rome, Vatican City Italy; 108 Department of Life and Reproduction Sciences, Section of Pediatrics, University of Verona, Verona, Italy; 109 Pediatric Allergy Unit, Severo Ochoa Universitary Hospital, Leganés, Spain; 110 Pediatric Allergy and Clinical Immunology Department, Hospital Sant Joan de Déu, University of Barcelona, Barcelona, Spain; 111 Division of Pneumology, Hospital Parc Taulí, Autonomous University of Barcelona, Barcelona, Spain; 112 Allergy Unit, Hospital de Terrassa, Barcelona, Spain; 113 Division of Allergy, University Department of Pediatrics, Pediatric Hospital Bambino Gesù, Rome, Vatican City Italy; 114 Human Microbiome Unit, Bambino Gesù Children Hospital, Rome, Italy; 115 Division of Allergy, University Department of Pediatrics, Pediatric Hospital Bambino Gesù, Rome, Vatican City Italy; 116 Division of Allergy, University Department of Pediatrics, Pediatric Hospital Bambino Gesù, Rome, Vatican City Italy; 117 Division of Infectious disease, University Department of Pediatrics, Pediatric Hospital Bambino Gesù, Rome, Vatican City Italy; 118 Department of Pharmacology, Poona College of Pharmacy, Bharati Videyapeeth Deemed University, Pune, India

## A1 Subcutaneous pollen immunotherapy by needleless device

### Giovanni Traina^1^, Rocco Luigi Valluzzi^2^, Vincenzo Fierro^2^, Carla Riccardi^2^, Maria Cristina Artesani^2^, Andrea De Vuono^3^, Alessandro Fiocchi^2^, Alberto G Martelli^1^

#### ^1^U.O.C Pediatria, Ospedale G. Salvini, Garbagnate Milanese, Milan, Italy; ^2^Division of Allergy, University Department of Pediatrics, Pediatric Hospital Bambino Gesù, Rome, Italy; ^3^Ufficio Statistica, Ospedale G. Salvini, Garbagnate Milanese, Milan, Italy

##### **Correspondence:** Giovanni Traina (giovannimariatraina@gmail.com)

BACKGROUND

In pediatric ages, sublingual immunotherapy (SLIT) allows allergic children to approach the inhaling desensitizing therapy. However, SLIT causes quite a few compliance problems and sometimes it is difficult to carry it out for the required long term. The alternative is subcutaneous immunotherapy (SCIT), commonly performed with a syringe. This technique hasn’t changed much in time and it is not without risks or discomfort. This has led to the search for alternative ways of vaccine administration, in order to reduce discomfort to children by improving compliance and diminishing the potential risks of adverse reactions.

OBJECTIVE

The study aimed to assess the patient’s perceived pain and the safety of a new way of administration of SCIT, with a needleless device.

METHODS

Children with grass or mite-induced allergic rhinoconjunctivitis and/or bronchial asthma were prescribed a glutaraldehyde-polymerized allergenic extract (Allergovac Polymeryzed®, Bial Aristegui, Italy). Each dose was divided in two parts: half injected with the traditional syringe (dose A), half in the other arm with a needleless device (Injex, Greytree, Ross on Wye, UK; dose B). Patients were blindfolded. The perceived pain and the difficulties of the procedure were registered on a dedicated VAS scale immediately (time 1) and 20 minutes after the first injection (time 20). Also, we evaluated the occurrence of occasional adverse events during such procedure and we assessed the perceived difficulty by the doctor administering with such method.

RESULTS

39 patients, aged 5-18 years, were recruited and assessed. All patients completed the study, which involved 468 grass pollen AIT SCIT doses, of which 234 with needleless device. At time 1, the use of the needleless device led to an 88% reduction of the pain perception average rate (from 16,8 to 1,97). The difficulty for the technician to make the inoculum was also analyzed (expressed by an increasing rate, from 0 to 4). In all 6 doses, for each child, the difficulty rate 2 relates to an almost constant number of patients, while the highest difficulty rates (grade 3 and grade 4) gradually decrease (until they disappear).

CONCLUSIONS

The vaccine administration with needleless device has various advantages and it ensures a better acceptance in comparison with administration by traditional SCIT. This is important in order to start the immunotherapy at an early stage, as it is recommended by the most recent studies, so that the natural evolution of the allergic disease can be immediately modified. The new technique ensures a higher safety both for the vaccinator and for the patient, with a better acceptance of the procedure by the patient and therefore a better and wider compliance to the vaccination schedules.

## A2 Clinical trial to evaluate the safety of camel milk intake in patients with cow’s milk protein allergy

### Luis Alberto Ríos, Christian R. Alcocer, Elsy Navarrete, Blanca Estela Del Rio Navarro, Victor Gonzalez, Berenice Velasco, Herberth J. Perez Aviles, Roberto Jose Fernandez, F. Cesar Pozo

#### Pediatric Allergy and Clinical Immunology, Hospital Infantil de México Federico Gómez, Mexico City, Mexico

##### **Correspondence:** Christian R. Alcocer (dr.craa@gmail.com)

BACKGROUND

Cow’s milk protein allergy (CMPA) affects from 0.6 to 0.9% of the general population, being a public health problem that mainly affects the pediatric population. The current treatment of CMPA involves the total elimination of the intake of this protein. Whenever a diet free of cow’s milk protein is prescribed, it must be nutritionally adequate. After the first year of life, camel milk has been proposed as an alternative to be ingested by patients suffering from CMPA by virtue of the difference in amino acid sequence with the cow’s milk protein, in addition it has a low amount of β-casein (Bos d8) and absence of β-lactoglobulin (Bos d5), as well as an adequate nutritional balance with components such as lactoferrin, immunoglobulins, lysozyme and vitamin C.

OBJECTIVE

To evaluate the safety of camel milk administration in patients with CMPA using double blind placebo controlled oral food challenge (DBPCOFC) methodology.

METHODS

A placebo-controlled, double-blind clinical trial was performed at the Hospital Infantil de México Federico Gómez (HIMFG). Inclusion was made in patients aged from 1 to 18 years with suspicion of CMPA, and a DBPCOFC to cow milk was performed to establish diagnosis. Only those in which the disease was corroborated by this method were randomized into to 2 groups:

- Administration of camel milk or
- Administration of amino acid formula

At the end of the corresponding challenge, the groups were crossed and the opposite challenge was performed previous 6 weeks of awaiting.

RESULTS

We included 49 patients with suspected cow’s milk protein allergy, and the diagnosis was established in 15 patients by DBPCOFC. None of them had adverse effects after the administration of camel milk during the oral challenge and 2 weeks later after daily intake.

CONCLUSIONS

This study demonstrates that camel milk is safe and adequately tolerated in patients older than 1 year old with CMPA and could be considered as an alternative, given the benefit of better taste, nutritional efficiency and possibly lower cost than other formulas.

## A3 Prevalence of anaphylaxis among children 3 to 18 years old in Kuwait

### Abdal Jabbar Farhan^1^, Hasan Arshad^2^, Ahmed Hussain^3^

#### ^1^Pediatric and Neonatology Department, New Mowasat Hospital, Salmiya, Kuwait; ^2^Faculty of Medicine, Southampton University, Southampton, United Kingdom; ^3^Amiri Hospital, Kuwait City, Kuwait

##### **Correspondence:** Abdal Jabbar Farhan (abdfarhan@yahoo.com)

BACKGROUND

Anaphylaxis is an acute though potentially life-threatening multisystem allergic reaction characterized by a rapid inception and progression following exposure to an allergen. Even though anaphylaxis has been on the rise for the past decade with reported rates of 350% increase among food induced and 230% increase among nonfood induced reactions, there is still paucity of data on the prevalence and outcome of anaphylaxis in various populations.

OBJECTIVE

This study aims to; (i) assess the lifetime prevalence of anaphylaxis among the paediatric population, (ii) identify the most at risk age, and (iii) evaluate the main trigger for the condition in children.

METHODS

A descriptive cross-sectional study with a multistage stratified cluster sampling of children aged three to eighteen was conducted in Kuwait. 2828 registered students of both genders and all nationalities in the selected governmental and private schools were sequentially recruited in the school years 2014-2015 and 2015-2016 over a period of 9 months to participate in the study.

RESULTS

The overall prevalence of anaphylaxis in the study population was 4.24 per 1000 with a 95% confidence interval of 1.85-6.64 per 1000 population. A total of 12 (0.42%) participants fulfilled all the criteria for diagnosing probable anaphylaxis, out of which 66.7% were diagnosed during the incidence by a medical physician. Food was the major trigger in 9 (75.0%) children overall, of which nuts was the most common (33.3%), followed equally by milk and egg (16.7%) and another 16.7% for other types of unspecified food. Medicine was the trigger in 2 (16.7%) of them and one was not sure of the trigger.

CONCLUSIONS

Anaphylaxis in children is a stimulating and challenging subject requiring targeted studies, starting with those assessing the prevalence of this emerging serious disease, to those highlighting the lack of availability, utilization and correct use of the treatment and the necessity of an emergency action plan.

## A4 Safety and efficacy of SLIT in children with an allergic asthma and a high level of IgE

### Olena Sharikadze^1^, Olena Okhotnikova^1^, Javier Alcover^2^, Diego Rodriguez^2^, Fernando Pineda^2^

#### ^1^Shupyk National Medical Academy of Postgraduate Education, Kyiv, Ukraine; ^2^Diater Laboratories, Madrid, Spain

##### **Correspondence:** Olena Sharikadze (shaolena@yandex.ru)

BACKGROUND

Although sublingual immunotherapy (SLIT) is increasingly used, data on the efficacy of SLIT in pediatric asthma remain limited.

OBJECTIVE

To evaluate the effectiveness of co-seasonal specific SLIT in children with allergic asthma and high IgE levels.

METHODS

Thirty children aged 3-17 years with allergic asthma and IgE > 600 IU/ml measured by IMMULITE 2000 XPi system (Siemens, Germany), sensitized to house dust mites and molds were included in a 2-year cohort study of the efficacy and safety of SLIT (Diater Laboratories, Spain) using standardized sublingual extracts containing house dust mites or molds (Alternaria alternata). Treatment efficacy was analyzed using the score of symptoms such as difficulty in nasal breathing, rhinorrhea, sneezing, itching of the nasal mucosa (upper palate) and discharge from the nose. Symptoms were measured before starting treatment, and at 4, 12 and 24 months after starting immunotherapy.

RESULTS

SLIT significantly improved asthma symptom scores (61% vs. control group), reduced nasal symptoms (45% vs. control group) and the use of rescue medications (20% vs. control group), and improved FEV1 (in children aged ≥ 5 years), but had no effect on IgE serum levels after 12 months of therapy. After the second year of SLIT, IgE serum levels were reduced by 10% vs. controls. In the SLIT group, predominantly local reactions were recorded in 32% of subjects in the first year of treatment and in 12% in the second year: 42% of children in the first year of SLIT and 18% in the second year had worsening of atopic dermatitis. No patient had a systemic reaction during therapy.

CONCLUSIONS

The use of sublingual standardized allergen extracts of house dust mites or molds (A. alternata) together with the clinical history (no history of anaphylaxis) make a correct diagnosis and the correct prescription of immunotherapy possible in children with high IgE levels. Our results show that co-seasonal SLIT in children with asthma and high IgE levels was safe and reduced the multiple symptom medication score.

## A5 Long-term outcomes following baked milk-containing diet for IgE-mediated milk allergy

### Ilan Dalal^1^, Jenny Weinbrand-Goichberg^2^, Shira Benor^3^, Menachem Rottem^4^, Shmuel Kivity^3^

#### ^1^Pediatric Allergy Unit, E. Wolfson Medical Center, Pediatric Department, E. Wolfson Medical Center, Sackler faculty of Medicine, Tel Aviv University, Tel Aviv, Israel; ^2^Pediatric Department, E. Wolfson Medical Center, Tel Aviv, Israel; ^3^Allergy and Clinical Immunology Unit, Tel Aviv Sourasky Medical Center, Sackler faculty of medicine, Tel Aviv University, Tel Aviv, Israel; ^4^Allergy Asthma and Immunology Unit, Emek Medical Center, Afula, Israel

##### **Correspondence:** Ilan Dalal (ilandalal@hotmail.com)

BACKGROUND

There is limited knowledge regarding long term follow-up of milk allergic patients who tolerate baked milk (BM) products.

OBJECTIVE

The aim of the study was to evaluate the long-term safety and efficacy of this intervention.

METHODS

Children with IgE-mediated milk allergy underwent an oral challenge with BM. Those who tolerated BM underwent baked cheese pizza (BC) challenge after 6 months. Six months later, a challenge with unheated milk was offered to patients who tolerated BC.

RESULTS

85 children (median 5.2 years; range 15 months to 15 years) were prospectively followed for a median of 29 months (range 15-50 months). Fifteen (18%) reacted to the initial BM challenge. Reactions were mild in most cases an only 3 had anaphylaxis. Among 70 (82%) children who initially tolerated BM challenge, 26 (37%) tolerated unheated milk at last follow-up, 16 (23%) tolerated BM/BC and 25 (36%) avoided all forms of milk despite successful initial BM/BC challenges. Another 3 patients (4%) were lost to follow-up. Predictive parameters of reactivity to initial BM challenge include age (median - 7; range 2.5-15 years vs. 3.5; range 1-14.5 years; P= 0.02), mild respiratory symptoms (67% vs. 26%, P<0.01) as part of the reported allergic reaction to milk, asthma (53% vs. 14.3%, P< 0.01) and a larger SPT to casein (median 14; range 8-28 mm vs. 6; range 0-18 mm; P<0.01).

CONCLUSIONS

For most of our patients, ingestion of BM was found to be safe and well tolerated. However, initial successful challenge with BM/BC does not always guarantee ongoing consumption of these products. Better predictors of response are needed for patient selection and long term outcomes.

## A6 Long-term follow-up of oral immunotherapy for food anaphylaxis

### Sakura Sato^1^, Noriyuki Yanagida^2^, Motohiro Ebisawa^1^

#### ^1^Department of Allergy, Clinical Research Center for Allergy and Rheumatology, Sagamihara National Hospital, Kanagawa, Japan; ^2^Department of Pediatrics, Sagamihara National Hospital, Kanagawa, Japan

##### **Correspondence:** Sakura Sato (s-satou@sagamihara-hosp.gr.jp)

BACKGROUND

Food anaphylaxis may be life-threatening.

OBJECTIVE

The aim of this study is to evaluate the efficacy and safety of oral immunotherapy (OIT) for treating anaphylaxis induced by allergy to hen’s egg (HE), cow’s milk (CM), wheat (W), or peanut (P), with a long term follow-up.

METHODS

Children with HE, CM, W, or P anaphylaxis, confirmed by the positive double-blind, placebo-controlled food challenge test, who received OIT were enrolled. Patients were admitted to the hospital in initial phase. Subsequently, the dose was slowly increased to a maintenance dose (HE, one heated-whole egg; CM [200 mL]; W, Udon noodle [200 g: 5.2g wheat protein]; P, peanut powder [3 g]) at home. Patients, who had ingested foods without any symptoms, underwent the final oral food challenge (OFC) after allergen avoidance for 2 weeks to confirm sustained unresponsiveness (SU). If the final OFC was positive, OIT was continued while dwindling the food intake interval (dwindling protocol). If patients did not have any symptoms, they repeated the final OFC. We evaluated the proportion of SU based on Kaplan-Meier analysis, and the safety of the dwindling protocol of OIT.

RESULTS

A total of 273 patients (HE; n = 84, CM; n = 114, W; n = 44, P; n = 31) were enrolled. Median follow-up time was 5.3 years, and 69 patients (25%) were lost to follow-up. The proportion of SU (%) was 87, 74, 64, and 57 in patients with P, W, HE, and CM anaphylaxes, respectively. The duration of time to achieve SU (years) was 1.5, 1.8, 2.2, and 5.0 in patients with P, HE, W, and CM anaphylaxes, respectively. One hundred nine patients continued the dwindling protocol of OIT. Of these patients, 33 patients (31%) underwent the second final OFC. Adverse reactions were present in 10% during the year after the first final OFC. Among them, mild symptoms were 83%, moderate symptoms 16%, and severe symptoms 1%, and 6 patients needed to use adrenaline auto-injector at home.

CONCLUSIONS

Efficacy of OIT for food anaphylaxis was different among food antigens, It was difficult to achieve SU during OIT for CM anaphylaxis even in long-term treatment. Even if patients who experience food anaphylaxis achieve desensitization by OIT, they have still a risk of anaphylaxis by dwindling the food intake frequency, and these patients need careful management during OIT.

## A7 Sublingual immunotherapy to house dust mites in pediatric patients with co-morbid asthma

### Tetiana Umanets^1^, Fernando Pineda^2^, Youriy Antipkin^1^, Vladyslava Barzylovich^1^, Volodymyr Lapshyn^1^

#### ^1^Institute of Pediatrics, Obstetrics and Gynecology, Kyiv, Ukraine; ^2^Diater Laboratories, Madrid, Spain

##### **Correspondence:** Tetiana Umanets (tetiana.umanets@gmail.com)

OBJECTIVE

To evaluate the effectiveness and safety of SLIT to HDM in children who have asthma with allergic rhinitis (AR) and atopic dermatitis (AD).

METHODS

A total of 61 children aged 6-7 y.o. with co-morbid asthma were evaluated and randomized to receive drugs with (n=32) or without (n=29) SLIT using the sublingual spray with 50% of Dermatophagoides pteronyssinus and 50% of Dermatophagoides farinae in a standardized extract (Diater Laboratories, Spain). The clinical scores, physical examination, lung function were assessed annually during 2 years of SLIT. Specific IgE against rDerp1, rDerp2, rDerp10, methacholine challenge were performed at the beginning and the end of the study.

RESULTS

The majority of patients had allergic asthma with AR (62, 3%) and AR, AD (37,7%). Sensitization to either rDerp1, rDerp2 or rDerp10 had respectively 13, 1%; 19, 7%; 0% and both rDerp1 and rDerp2 were detected in 67, 2% of children. During the first year of treatment there were no significant differences in the clinical symptoms scores of AR and AD, but symptoms of asthma were improved in 65, 6% SLIT group vs control group. A decrease in the clinical symptom scores of asthma, AR and AD was observed in 84, 4%, 65, 6% and 66, 6% of children after 2 years of SLIT (p<0.05 vs control group). There was no the significant deviations of FEV 1, sIgE levels to HDM proteins during the study. The number of children with positive methacholine challenge results decreased after 2 years specifically in the SLIT group. SLIT was well tolerated with only local reaction reported.

CONCLUSIONS

HDM SLIT appears to be effective and safe in children with co-morbid asthma and can simultaneously treat other target organs involved in the allergic process. To achieve good control of asthma and allergic co-morbidities, the use of more prolonged SLIT therapy is recommended.

## A8 Safety and efficacy of sublingual bacterial inactivated whole-cell suspension in adolescents with chronic rhinosinusitis: Novel strategy of personalized approach?

### Tetiana Umanets^1^, Mykola Umanets^2^, Sergey Yuriev^3^, Fernando Pineda^4^, Diego Rodriguez^4^, Javier Alcover^4^

#### ^1^Institute of Pediatrics, Obstetrics and Gynecology, Kyiv, Ukraine; ^2^Heart Institute, Kyiv, Ukraine; ^3^Ukrainian School of Molecular Allergy and Immunology, Kyiv, Ukraine; ^4^Diater Laboratories, Madrid, Spain

##### **Correspondence:** Tetiana Umanets (tetiana.umanets@gmail.com)

OBJECTIVE

To assess safety and efficacy of sublingual bacterial phenol inactivated whole-cell suspension (OM1, Diater Laboratories, Spain) in adolescents with CRS without nasal polyps (CRSsNP).

METHODS

39 patients aged 13- 16 were enrolled in this study. They were randomized to receive the sublingual bacterial phenol inactivated whole-cell suspension of the following bacteria (OM1): *Staphylococcus aureus*, *β-hemophilic streptococcus*, *Streptococcus pneumoniae*, *Haemophilus influenzae* (n=19) or the treatment according to World Allergy Organization recommendation (n=20). The clinical control of CRS, visual analogue scale (VAS) score, number of antibiotics courses and bacterial cultures were performed at pre-treatment and 12-month follow-up visits. The bacterial counts graded by the laboratory from 103 to 105 CFUs/mL were analyzed.

RESULTS

The majority of patients had partly controlled 34 (87,2%) or uncontrolled CRSsNP 5 (12,8%). According to VAS score included patients were divided on moderate 30 (76, 9%) and severe 9 (23, 1%) CRSsNP. *Staphylococcus aureus* colonization was noted in 31 (79, 5%) patients, both *Staphylococcus aureus* and *Streptococcus pneumoniae* in 6 (15,4%), both *Staphylococcus aureus* and *Haemophilus influenzae* in 2 (5,1%). There was a significant decrease in VAS score and increase in controlled CRSsNP particularly in OM group (p<0.01). The number of antibiotics courses decreased in 33 (84,6%) patients of OM group and only in 12 (30,7%) of control group. Reduction of the bacterial count in *Streptococcus pneumoniae*-colonized and *Staphylococcus aureus*-colonized adolescents was significant after the OM sublingual therapy and was non-significant after use of standard drugs. No adverse events related to OM therapy were observed.

CONCLUSIONS

The sublingual bacterial inactivated whole-cell suspension appears to be effective and safe in adolescents with CRSsNP and therefore can be considered an additional treatment option in patients with resistances to standard treatment’s approach.

## A9 How family practitioners are ideally positioned to perform preschool screening for the silent epidemic of mouth breathing developing from chronic allergic rhinitis

### Suzan Bekir^1,2^, Tobias Pincock^1,2^

#### ^1^Collective.care Allergy Clinic, Sydney, Australia; ^2^Australian Allergy Centre, Sydney, Australia

##### **Correspondence:** Suzan Bekir (drsuzanbekir@hotmail.com)

BACKGROUND

The vast majority of health care professionals remain unaware of the negative effects of mouth breathing on normal facial growth and physiological health in children. By adding a “mouth breathing” screen to existing 4 Year Healthy Kids checks, we hypothesise that general practitioners (GPs) can identify mouth breathing quickly preventing craniofacial growth abnormalities, dental and orthodontic problems, poor posture, reduced exercise capacity, sleep disordered breathing, fatigue, worsening of asthma, dry mouth, reduced saliva and halitosis and below potential academic performance. Typically adenoidal hypertrophy and allergic rhinitis are the commonest cause of mouth breathing in children and allergy is a potential modifiable risk factor.

OBJECTIVE

To evaluate the possible role of GPs in performing comprehensive mouth breathing screening tests at preschool age to identify mouth breathing and prevent chronic complications of allergic rhinitis.

METHODS

Create an evidence-based algorithm for mouth breathing to be used by GPs as a clinical pathway which could successfully identify chronic complications of allergic rhinitis and sleep disordered breathing. Determine whether after completing the 6 months accredited GP training program in Allergy/ENT GPs could successfully learn and apply skills such as nasoendoscopy, rhinomanometry and allergy skin prick testing to successfully perform the clinical pathway.

RESULTS

All GPs enrolled in the training program were successfully trained to perform the mouth breathing clinical pathway which included paediatric focussed allergy and ENT history, examination of oral cavity and nasopharyngeal airway assessment with nasoendoscopy, identifying allergic rhinitis and co-ordinating a multidisciplinary approach to mouth breathing involving ENT for adenoidectomy and/or adenotonsillectomy as well as allied health involvement such as orthodontic and myofunctional therapy and speech pathology.

CONCLUSION

Mouth breathing is a pathology and not a variant of normal and warrants a comprehensive examination identifying allergic rhinitis, adenoidal hypertrophy and/or functional mouth breathing. GPs are in the best position to screen for mouth breathing when performing routine oralchecks at the 4 Year Healthy Kids Check. GPs were able to identify causes of mouth breathing as well as comprehensively treat and co-ordinate care with ENT, allergists, orthodontists and oromyofacial therapists.

## A10 A dust mite (HDM) lower dose intradermal immunotherapy (IDIT): A cost-effective approach for allergy rhinitis patients in underserved population

### Alberto Vieira Hernandez^1^, Arnaldo Capriles Hulett^2^, Mario Sánchez Borges^3^, Fabiola Fabiano^4^, Carlos Albarran^2^

#### ^1^Hospital de Emergencias de Naiguata, Naiguata, Venezuela; ^2^Departamento de Alergologia, Hospital de San Juan De Dios, Caracas, Venezuela; ^3^Departamento de Pediatria, Centro Medico Docente La Trinidad, Caracas, Venezuela; ^4^Laboratorio Torre Caracas, Caracas, Venezuela

##### **Correspondence:** Alberto Vieira Hernandez (albertovieiraher@gmail.com)

BACKGROUND

High Dose Subcutaneous Immunotherapy (SCIT) has proven effectiveness; however, due to its cost allergy vaccines are not available for low-income patients.

OBJECTIVE

This fact encouraged us to perform a “proof-of-concept” study of a HDM lower weekly dose to be administered intradermally (IDIT, containing Dermatophagoides pteronyssinnus/Dermatophagoides farinae (Dp/Df) and Blomia tropicalis (Bt)) in pediatric allergic rhinitis patients symptomatic on exposure to house dust.

METHODS

Symptomatic subjects with no previous immunotherapy (SLIT/ SCIT) use were selected. All subjects had positive prick skin tests (> 3 mm wheal) to a Dp/Df mixture (10.000 AU/ml) and Bt (30.000 DBU/ml) and negative prick skin tests to dog and cat epithelia, cockroach and grasses. No tobacco exposure or pets at home were allowed. All children received a 0.05 cc weekly IDIT dose containing 83 AU of Dp/Df mixture (0.5 micrograms HDM major allergens) and 250 DBU of Bt. All patients filled out a daily Symptom Score and a Visual Analog Scale (VAS) chart for 2 weeks before IDIT treatment was commenced, and the day before the weekly dose administration, for 12 weeks. Serial dilutions prick skin tests (SDSPT) were performed before and after the IDIT treatment. Statistical comparisons of quantitative variables (mean values) were made between total nasal symptom scores (TNSS), VAS, and wheal size (SDSPT) before and after treatment, employing the Mann-Whitney test with a P<0.05 considered statistically significant.

RESULTS

Eight patients completed the study. TNSS and VAS scores were found to be significantly lower when compared to pre-treatment week; also, SDSPT wheal sizes were significantly lower on post-treatment evaluation.

CONCLUSIONS

This “proof of concept” study shows significant improvement in symptoms of allergic rhinitis in patients that received a 14 fold less weekly IDIT dose (0.5 μg), for 12 weeks, when compared to the reported SCIT effective maintenance dose of 0.5cc (7 μg of HDM Major Allergens). An immunologic response, as evidenced by SDSPT, as a correlate of improvement, was also observed. A lower weekly dose IDIT has a favorable clinical impact at a fraction of the conventional SCIT dose; it might be considered as a cost-effective alternative treatment for developing countries.

## A11 Comparison of food allergy prevalence among Indian infants in non metro city of India, 2009 versus 2016

### Rohit Goyal^1^, Shilpa Gupta^2^, Garg Gaurav^3^

#### ^1^Max Super Speciality Hospital, Bathinda, India; ^2^Batra Hospital, New Delhi, India; ^3^Deen Dayal Upadhyay Hospital, New Delhi, India

##### **Correspondence:** Rohit Goyal (rohit.goyal@maxhealthcare.com)

BACKGROUND

Food allergy prevalence is increasing in developed countries, but these results have not yet been verified in developing countries, especially in india.

OBJECTIVE

Our aim was to determine whether the prevalence and characteristics of food allergy have changed over the last 7 years in Bathinda, India.

METHODS

Two cross-sectional studies were performed, 7 years apart (2009 and 2016) using the same diagnostic methods in the same age group (0–24 months) of the same hospital. A total of 401 infants were randomly selected for the present study. Food allergy was confirmed by food challenge. SPSS 15.0 was used to analyze the difference in prevalence.

RESULTS

Food allergy prevalence increased significantly from 3.5% in 2009 to 7.7% in 2016 (P= 0.017). The prevalence of a positive skin-prick-test response was also increased (from 9.9% to 18%; P= 0.002). Egg and cow’s milk were still the most common food allergens, which cause skin and gastrointestinal symptoms in most infants.

CONCLUSIONS

This is the first study in India to indicate time trends in food allergy prevalence and characteristics. Our data show that in the 7-year period from 2009 to 2016, the prevalence of food allergy seems to have increased in India.

## A12 Decreased exacerbations and hospitalizations in adolescents enrolled in PROSPERO (Prospective observational study to evaluate predictors of clinical effectiveness in response to omalizumab)

### Allan T Luskin^1^, Noelle M Griffin^2^, Amy Wagelie-Steffen^3^, Benjamin L. Trzaskoma^2^, Susan L Limb^2^, William W Busse^4^, Robert S Zeiger^5^, Erika Gonzalez-Reyes^6^, Thomas B Casale^7^, Bradley E Chipps^8^

#### ^1^HealthyAirways, Madison, WI, USA; ^2^Genentech, Inc., South San Francisco, CA, USA; ^3^Allergy Associates of Tucson, P.C., Tucson, AZ, USA; ^4^University of Wisconsin School of Medicine and Public Health, Madison, WI, USA; ^5^Deptartment of Allergy and Research and Evaluation, Kaiser Permanente Southern California, San Diego, CA, USA; ^6^The Children’s Hospital of San Antonio, Baylor College of Medicine, San Antonio, TX, USA; ^7^Division of Allergy and Immunology, University of South Florida, Tampa, FL, USA; ^8^Capital Allergy and Respiratory Disease Center, Sacramento, CA, USA

##### **Correspondence:** Bradley E Chipps (bchipps@capitalallergy.com)

BACKGROUND

Prospective real-world data are important to supplement clinical trial data in heterogeneous diseases like asthma. Data for adolescents are essential, as they are often under-represented in clinical trials.

METHODS

69 patients aged between 12-17 years old with asthma, identified as omalizumab candidates by their treating physicians and with access to omalizumab through insurance or other funding, were enrolled as part of the PROSPERO study. Patients were followed for a maximum of 48 weeks. At baseline and throughout the study, asthma-related healthcare utilization and exacerbations, were recorded. Asthma control was recorded monthly using the Asthma Control Test (ACT). Spirometry was performed and biomarkers (FeNO, blood eosinophils) were collected at baseline, 6 months and end of the study.

RESULTS

59 (86%) adolescents completed 48 weeks of the study; mean (SD) age 14.0 (1.69) years, primarily male (64%) and white (58%), with 33% Black or African American. On average, adolescents reported 2.8 exacerbations (67% with ≥ 2 exacerbations) that required oral corticosteroid use, ED visit or hospitalization in the 12 months prior to enrollment. 28% reported ≥ 1 asthma related hospitalization in the prior year. At month 12, a mean rate of 0.46 exacerbations per year was observed, representing an 84% reduction from baseline. 11.5% of patients reported ≥ 2 exacerbations and 4% reported ≥ 1 hospitalization, an 83% and 86% reduction from baseline. Mean (SD) baseline FEV1 percentage predicted was 88% (17) and improved on average by 8% to 95% (20), an absolute improvement of 160mLs compared with baseline. 71.6% (48/67) of adolescent patients with baseline ACT data reported poorly controlled asthma at baseline based on ACT score of <20. At the study’s conclusion, 68.8% of these poorly controlled patients were controlled (ACT ≥ 20). Baseline mean (SD) FeNO levels were 38.4 (39.4) ppb and mean (SD) eosinophils were 316 (210) cells/μL, with 54.5% of patients having eosinophils ≤ 300 cells/μL. Median (interquartile range) total IgE levels were 507 (960.7) IU/mL. At the end of study, mean (SD) FeNO levels decreased by 27% to 29.2 (28.1) ppb and mean eosinophils (SD) decreased by 34% to 209 (151) cells/μL. Adverse events were consistent with the safety profile described in the current product label.

CONCLUSIONS

In a real-world setting, adolescents treated with omalizumab had significant improvements in asthma control as demonstrated by decreased exacerbations and hospitalizations, and improved ACT scores compared with baseline.

## A13 Comparison of infantile allergic disease prevalence with a 12 year interval (3rd report)

### Chizuko Sugizaki, Fumiko Goto, Sakura Sato, Noriyuki Yanagida, Motohiro Ebisawa

#### Department of Allergy, Clinical Research Center for Allergy and Rheumatology, Sagamihara National Hospital, Sagamihara, Japan

##### **Correspondence:** Chizuko Sugizaki (c-sugizaki@sagamihara-hosp.gr.jp)

OBJECTIVE

We had followed up a birth cohort in Sagamihara-city to clarify profiles of allergic diseases during the first 7 years of life from 2002. We began to follow the same cohort again from 2014 with a 12 year interval. The purpose of the current survey is to clarify changes in prevalence of infantile allergic diseases and environmental factors, compared with data from 2002.

METHODS

Using the mass medical examination system at the age of 4 months (m), we obtained basic profile information on subjects from January 1, 2014 to December 31, 2014. We have followed up the subjects whose parents had agreed to participate in this study at the ages of 8 and 12 m. We utilized the same questionnaire on atopic dermatitis (AD) and food allergy (FA) that was mailed in 2002.

RESULTS

The number of subjects in the 2014 study was 4,646 with a recovery rate of 83.9% (2002 study: 5,247 with a recovery rate of 88.5%). We analyzed cases that answered 3 times at 4, 8, 12 m old, except for incomplete answers. There were 2,529 cases in the 2014 study and compared the results with 3,196 cases in the 2002 study. The incidence of eczema with suspected AD was 19.6%/ 17.0%/ 15.5% at 4/8/12m, respectively (26.3%/ 18.7%/ 13.0% in 2002). The rate of diagnosis of AD was 1.0%/ 2.6%/ 3.1% (1.2%/ 2.8%/ 3.2% in 2002). “Reported FA” includes a case with eliminating foods or using special milk due to FA. The rate of “Reported FA” was 8.8%/ 18.8%/ 19.7% (6.6%/ 19.1%/ 14.6% in 2002). The rate of diagnosis of FA was 0.1%/ 1.2%/ 1.5% (0.1%/ 2.3%/ 2.7% in 2002). We detected significant changes of environmentaL factors in 4 m old infants with a 12 year interval. The rate of positive family history with allergic disease, the rate of raising pets and the rate of breast feeding only cases increased. The average of weight, the rate of indirect cigarette exposure, the rate of artificial feeding only cases and the rate of first child decreased.

CONCLUSIONS

There was no statistically significant difference between the 2002 and 2014 studies regarding the rate of diagnosis of AD. “Reported FA” cases increased at 4 and 12 m significantly; on the other hand, diagnosis of FA was reduced significantly at 8 and 12 m.

## A14 Rush oral immunotherapy is a safe and effective treatment for children with severe wheat allergy

### Akiko Yamaide^1^, Kanako Mitsunaga^1^, Minako Tomiita^1^, Akira Hoshioka^1^, Naoki Shimojo^2^

#### ^1^Department of Allergy and Rheumatology, Chiba Children’s Hospital, Chiba, Japan; ^2^Department of Pediatrics, Chiba University Graduate School of Medicine, Chiba, Japan

##### **Correspondence:** Akiko Yamaide (yamaide@abox22.so-net.ne.jp)

BACKGROUND

Wheat is the third most common cause of food allergy in Japanese children. But there are few reports of oral immunotherapy (OIT) for wheat allergy.

OBJECTIVE

Here we report about our rush OIT of wheat for school-age children of severe wheat allergy with anaphylaxis.

METHODS

Subjects were 6 boys and 1 girl (6 to 10 years of age) of wheat allergy. Their threshold doses of wheat inducing allergic reactions were between 0.25 and 10 g of Japanese traditional wheat noodle (UDON; 1g of UDON contains 25mg of wheat protein). The wheat specific IgE value was 5.46-100< UA/ml.

All subjects were hospitalized during rush OIT. Intramuscular adrenaline, oral antihistaminics and corticosteroid, oxygen, and bronchodilator for inhalation were ready to be used at the bedside at all times. The initial dose for each subjects was set at approximately one-eighth to one-half of the threshold dose for that subjects. Thereafter, doses were increased by 1.5 times and administered twice a day at 1 hour interval. The goal of rush OIT was 80 to 180 g of wheat noodle according to their age and families’ choice. For preventive therapy, DSCG was used in three subjects and leukotriene receptor antagonist was used in six subjects, and antihistaminic agent was used in all subjects.

RESULTS

All subjects acquired desensitization to target dose of rush OIT (80 to 180 g of wheat noodle). It took 2 to 4 weeks. No serious reactions which needed epinephrine injection were observed. Medication (2.6 antihistaminic agents/person, 1.0 corticosteroid medicine/person) and inhalation treatment (0.7 times/person) were required for the treatment of adverse reaction accompanying rush OIT (rash, cough, wheezing, stomachache, diarrhea, etc). All subjects continued OIT after discharge from hospitals.

CONCLUSIONS

Our rush OIT was a safe and effective treatment for severe wheat allergy.

## A15 Cow’s milk allergy in cystic fibrosis children

### Liviu L Pop^1,3^, Ioana-Mihaela Ciucǎ^1,3^, Liviu Tǎmaş^2^, Marilena Lazarescu^3^, Corina Pienar^1^

#### ^1^Department of Pediatrics II, Victor Babeş University of Medicine and Pharmacy Timisora, Timisoara, Romania; ^2^Department of Biochemistry, Victor Babeş University of Medicine and Pharmacy Timisora, Timisoara, Romania; ^3^National Cystic Fibrosis Center, Timisoara, Romania

##### **Correspondence:** Liviu L Pop (liviupop63@yahoo.com)

BACKGROUND

In cystic fibrosis (CF) an important feature is the pancreatic insufficiency expressed by steatorheea; in some cases, despite enzyme supplementation the chronic diarrhea is poorly controlled.

OBJECTIVE

Considering the increasing frequency of cow’s milk allergy (CMA), we consider it interesting to find the prevalence of cow’s milk allergy among patients with CF.

METHODS

In one year, sixty seven children with CF, aged 1 month - 3 years (19 infants), followed in our center were observed for CMA. Cow’s milk allergy was suspected in the presence of specific clinical manifestations (gastrointestinal symptoms, cutaneous signs, respiratory features), or suggestive medical history (failure to thrive, colics etc). In addition CMA-specific IgE (α- and β-lactoglobulin, casein) and diagnostic elimination tests were performed.

RESULTS

Among infants, CMA was diagnosed in 47.36% (9 patients) of children, predominantly in pancreatic insufficient children (77% -CF infant with pancreatic insufficiency). Toddlers (1-3 years) were diagnosed in a smaller percent, only 16.6% (8 children) proved to have CMA, although in more than 35% of toddlers, a positive history for CMA diagnosis was found. CF patients with pancreatic insufficiency associated more frequently CMA (88.23%) than cystic fibrosis patients pancreatic-sufficient. The prevalence of cow’s milk allergy was important, cumulating a 25.37% of CF patients.

CONCLUSIONS

Cow’s milk allergy was frequently found in CF children, especially associated with pancreatic insufficiency. The “combined” enteropathy could influence the disease’s outcome and should be considered especially in persistent diarrhea of CF children with correct enzyme supplementation.

## A16 Serum zonulin levels are higher in pediatric allergic patients than that in healthy children

### Fumiya Yamaide, Bahrul Fikri, Hironori Sato, Naoki Shimojo

#### Department of Pediatrics, Graduate School of Medicine, Chiba University, Chiba, Japan

##### **Correspondence:** Fumiya Yamaide (fyamaide@chiba-u.jp)

BACKGROUND

Zonulin, an epithelial tight-junction regulator, plays an important role in the regulation of epithelial barrier function and was reported to play a pathogenic role in celiac disease and other chronic inflammatory diseases. Allergic sensitizations are the most important risk factors for development of allergic diseases. Allergen penetration through epithelium to the body is important for allergic sensitizations (cf.percutaneous sensitization to food allergens). Because allergen sensitization and chronic inflammation are important in the pathogenesis of allergic diseases, zonulin is suggested to paly important roles in allergic diseases. But there are few reports about zonulin levels in pediatric allergic patients.

OBJECTIVE

The aim of this study was to investigate the relationship between serum zonulin levels and allergic diseases in children.

METHODS

The study included allergic children and healthy children who were of similar age and gender distribution (8 healthy infants and 8 allergic infants, and 8 healthy school-age children and 8 school-age allergic children). The serum zonulin level was measured by ELISA.

RESULTS

In this study, total IgE levels in all allergic patients and healthy children were above normal levels and below measurable limits, respectively. All allergic infants had atopic dermatitis and food allergy, and allergic school-age children had food allergy or bronchial asthma or both. In infants, mean zonulin levels were 30.5 ng/ml in allergic patients and 17.8 ng/ml (p=0.0028). In school-children, mean zonulin levels were 29.8 ng/ml in allergic patients and 8.7 ng/ml (p<0.0001). We also investigated whether zonulin levels are different among allergic diseases.

CONCLUSION

In infants and school-age children, serum zonulin levels are higher in allergic children than that in healthy children.

## A17 Possible application of a pack cooking that helps food allergic patients at the time of disaster

### Naoko Okishima^1^, Miyabi Kobayashi^1^, Mizuki Takai^1^, Kotarou Nishigata^1^, Ryou Yoda^1^, Yu-ta Oana^1^, Chifu Kajiwara^1^, Moe Shimodaira^1^, Tomoka Suzuki^1^, Hiromi Iizawa^2^, Koji Kamijo^3^

#### ^1^Department of Health and Nutritional Science, Matsumoto University, Matsumoto, Japan; ^2^Health Support Station, Matsumoto University, Matsumoto, Japan; ^3^JDA-DAT, Matsumoto, Japan

##### **Correspondence:** Naoko Okishima (naoko.okishima@matsu.ac.jp)

BACKGROUND

The Great East Japan Earthquake on March 11, 2011 brought extensive damage to northeast Japan. Essential utilities were damaged and a lot of people were forced to evacuate. They had to stay at the temporary evacuation shelter and take ration foods. However, refugees with food allergy could not eat the foods because the ration foods were supplied without considering food allergy. According to the reports, the caregivers of children with food allergy were confronted with difficulties to get allergen-free foods, and actually some children developed allergic symptoms.

OBJECTIVE

Pack cooking attracted lots of attention in Japan after The Great East Japan Earthquake, because the cooking method enables cooking by portable gas stove and with less water. Since the ingredients are changeable in each bag, pack cooking is convenient to make individual foods. In this report, we emphasize the usefulness of pack cooking under emergency and propose cooking recipes. Moreover, we show that the allergen cross-contamination hardly occurred among the bags by the ELISA analyses.

METHODS

Recipes of pack cooking without 7 specified raw materials for food allergy in Japan (eggs, milk, wheat, peanuts, buckwheat, shrimps and crabs) were designed. All recipes were cooked at the test kitchen of Matsumoto University. Ingredients were thinly sliced and put into a plastic bag made of polyethylene with seasonings. The top of the each bag was twisted and closed tightly. Then the bag was cooked in boiled water for about 30 minutes until the ingredients were fully cooked. To estimate the contamination of the allergens during cooking, the bags contained 5 food allergens; eggs, wheat, buckwheat, shrimps and crabs were boiled together with the allergen-free bags containing some ingredients. The amount of allergens was assessed by the commercially available ELISA kits for each allergen.

RESULTS

We will show 20 recipes for the allergen-free pack cooking menu at the session. The ELISA analyses revealed that no wheat allergen was detected in the ingredients bags at all. Concerning the other 4 allergens, little contamination was observed. Different analysis of methods such as the western blot or PCR were required to exclude the non-specific reaction. Subsequently, we are developing the weaning food’s recipes because it is also very difficult to get foods for babies at the time of disaster. We will show the latest data on site.

## A18 Study on seasonal variation of airborne fungal spores in urban and rural areas of West Bengal, India, and a search for novel allergens from *Aspergillus terreus*

### Bijoya Karmakar^1^, Swati Gupta Bhattacharya^2^

#### ^1^Senior Research Fellow, Bose Institute, Kolkata, India; ^2^Senior Professor, Bose Institute, Kolkata, India

##### **Correspondence:** Bijoya Karmakar (bijoyak5@gmail.com)

BACKGROUND

Airborne fungal spores are one of the most important allergen sources worldwide.

OBJECTIVE

The present study aimed to investigate the seasonal variations of airborne fungal spores found in indoor and outdoor environments of urban and rural areas of West Bengal and to determine allergenic potency of *Aspergillus terreus* through immuno-biochemical and *in silico* methods.

METHODS

Aerobiological monitoring was performed using Andersen and Burkard Personal sampler for trapping viable and non-viable spores from the air of Kolkata (Urban) and Habra (Rural), West Bengal, India. A questionnaire survey and hospitalization data were collected from the Habra State General Hospital. Protein from *A. terreus*, dominant aero-spore in the studied area, was extracted for determining its allergenecity by SPT, ELISA and histamine release assay. Protein profile of *A. terreus* was analyzed by 1D and 2D gel electrophoresis, followed by the immunoblotting with 15 individual sensitized patients’ sera and identification of allergenic spots through MASCOT analysis. One of the allergens has been purified for further biochemical study, followed by *in silico* allerginicity assessment using bioinformatics tools.

RESULTS

The comparative study revealed that the rural area showed maximum spore load as compared to the urban area. The survey and hospitalization records (ARI, COPD patients) were found to be positively correlated with the total spore load. Histamine release, specific IgE and skin sensitivity were also found in higher amount in susceptible patients. 2D gel blot revealed sixteen spots as immuno-reactive upon immunoblotting with sensitized patients’ sera. MALDI TOF/TOF analysis led to the identification of 3 allergens as BMH2 (similarity with 14-3-3 protein), triose phosphate isomerise and glutathione S-transferase. Purification of a phosphotriose isomerase protein was carried on due to its highest IgE reactivity, followed by the prediction of their epitopes using allergen prediction servers.

CONCLUSIONS

This study will help to determine the presence of the diversified fungal species and their total concentrations in the air of the sampling site. In West Bengal, *A. terreus* is the commonest among fungi and three allergens have been reported from this fungus.

## A19 Epidemiology of food allergy in Czech children, results of DAFALL registry

### Simona Blohlávková^1,2^, Eliška Kopelentová^3,4^, Petr Víšek^5^, Jakub Štádler^1^, Ivana Šetinová^6^, Jana Novobílská^7^

#### ^1^Immuno-flow, s.r.o., Prague, Czech Republic; ^2^Medical Faculty, Charles University, Pilsen, Czech republic; ^3^Allergology Department, Kolín Hospital, Kolín, Czech Republic; ^4^Allergology and Immunology Department, Faculty Hospital Motol, Prague, Czech Republic; ^5^Allergology and Immunology Department, Litomyšl, Czech Republic; ^6^Immunia, s.r.o., Prague, Czech Republic; ^7^Alergollogy Novobílská, Vratimov, Czech Republic

##### **Correspondence:** Simona Blohlávková (simona.belohlavkova@seznam.cz)

BACKGROUND

Occurence of food allergy has significantly risen in recent decades. Its prevalence is 6-8% in children. There are some differencies in incidence of types of food allergy in different geographical areas. Only limited data are known about the most common causes of food allergy in Czech Republic.

OBJECTIVE

The aim of the study is to gather epidemiological data describing patients with food allergy in the Czech Republic.

METHODS

DAFALL- Database of Food Allergies - is an electronic registry founded in October 2014. The most common triggers of food reactions, threshold doses, processing of food allergens, laboratory test results including component resolved diagnosis, skin prick tests and food challenges results as well as allergology history of the patients were evaluated.

RESULTS

During the first 28 months until February, 2017, 1,276 patients were enrolled from more than 30 collaborating allergology outpatient clinics; 49% children under age of 6 years (n=624), 26,5% children aged 6-18 years (n=337) and 24,5% adults (n=315). In children under 1 year of age, cow’s milk is the most frequent food allergen. In 86% of cases, first symptoms of milk allergy were recorded below age of 7 months and in 60% of cases were noted in fully breast-fed kids. About 60% of milk reactions were non-IgE mediated, with no prove of any positivity in skin prick tests and/or specific IgE against milk. Most common triggers of allergy in children between 1 and 6 years of age were milk (40%), egg (34%), tree nuts (22%), peanut (17%) and fruits (11%). In patients older than 6 years, significant allergens are tree nuts (hazelnut, walnut, almond), fruits (apple, peach, kiwi), vegetables, peanut and seeds, mainly the sesame seed and poppy seed. The poppy seed is one of the typical Czech food allergens in all ages. On the other hand, we found only very limited number of patients with adverse reactions to soy.

CONCLUSIONS

DAFALL is the first project describing relevant data on food allergy in the Czech population. The projected period for data collection is 3 years and we expect to enroll 1500-2000 patients. More than 70% of patients enrolled are children, so we will be able to give a detailed description of the Czech food allergic children.

## A20 Long-term safety and efficacy of perinatal probiotic intervention: evidence from a follow up study

### Krisa Lundelin^1^, Seppo Salminen^1,2^, Erika Isolauri^1^

#### ^1^Department of Paediatrics, Turku University Hospital, Turku, Finland; ^2^Functional Foods Forum, University of Turku, Turku, Finland

##### **Correspondence:** Krisa Lundelin (kristalundelin@hotmail.com)

BACKGROUND

Societies worldwide are faced with a progressive increase in immune-mediated health problems such as allergic, autoimmune and inflammatory diseases, as well as obesity. Perinatal administration of specific probiotic bacteria is an attractive approach in reducing the risk of these conditions, but long-term efficacy and safety data are lacking.

OBJECTIVE

The aim here was to evaluate the clinical benefit and long-term safety of specific probiotics administered during the perinatal period.

METHODS

The probiotic strains used were *Lactobacillus rhamnosus* GG (LGG), *Bifidobacterium lactis* Bb-12, *Lactobacillus paracasei* ST11 and *Bifidobacterium longum* BL999. The children involved have subsequently undergone prospective long-term follow-up. In addition to physical examination, data were collected by structured questionnaires on non-communicable diseases and continued probiotic use.

RESULTS

Altogether, 303 mother-infant pairs were included in the analysis. Allergic disease was the most common chronic condition with an overall prevalence of 51.2%. It consisted in allergic rhinitis (prevalence 42.3%), atopic eczema (18.9%), asthma (12.7%) and food allergy (1.4%). In children given specific probiotics or probiotic combinations perinatally there was a tendency to a decreased prevalence of allergic disease, OR 0.67, 95% CI 0.43-1.06, p=0.09. In those receiving LGG perinatally the decrease in the prevalence of allergic disease (59/133, 44.4%), as compared to placebo (79/140, 56.4%), was statistically significant, OR 0.62, 95% CI 0.38-0.99, p=0.047. The prevalence of asthma in the children receiving probiotics perinatally, 9.3%, was not statistically different from that in children given placebo, 15.8%; (OR 0.55, 95% CI 0.24-1.25, p=0.15), nor was the prevalence of asthma in children receiving perinatally LGG alone or in combination (10/96, 10.4%), statistically different from that in children given placebo (18/114, 15.8%); (OR 0.62, 95% CI 0.27-1.42, p=0.26).

CONCLUSIONS

Perinatal probiotic administration is safe in long-term follow-up. Children receiving *Lactobacillus rhamnosus* GG perinatally tended to have decreased allergy prevalence.

## A21 A case of an allergic reaction to *Colocasia antiquorum,* or eddoe

### Tracy Pitt^1^, Tamar Flanders^2^

#### ^1^Department of Paediatrics, Humber River Hospital, Toronto, ON, Canada, and Deptartment of Paediatrics, Queen’s University, Kingston, ON, Canada; ^2^Midtown Paediatrics, Toronto, ON, Canada

##### **Correspondence:** Tracy Pitt (tracypitt@hotmail.com)

OBJECTIVE

*Colocasia antiquorum,* or eddoe, is a tropical vegetable found predominantly in China, Japan and the West Indies. It is considered a more flavourful alternative to potato or yams. We report one of the first cases of a patient with an allergic reaction to eddoe.

METHODS

I.R is a 10-month-old female with atopic dermatitis, who was referred to a local allergist for an episode of emesis almost immediately after ingestion of eddoe on two separate occasions. Milk was ingested with eddoe on one of these occasions. She also reports swollen lips, urticaria and vomiting within 3 hours of ingestion of eggs. Skin testing was performed to fresh peanut, milk extract, fresh milk, egg extract, fresh egg and fresh eddoe.

RESULTS

Skin testing to fresh peanut, milk extract, fresh milk, egg extract, fresh egg and fresh eddoe was positive to fresh egg (16mm) and fresh eddoe (5mm). The patient was instructed to avoid eggs and eddoes and an Epi pen Jr was prescribed.

CONCLUSIONS

To our knowledge, this is one of the first case reports of an allergic reaction to *Colocasia Antiquorum*, or eddoe. Further research is needed to characterize the allergenic components of this vegetable.

CONSENT TO PUBLISH

Consent to publish was obtained from the parents of the patient in this study.

## A22 Efficacy and safety of Beltavac Polimerizado administered in children between 3 and 11

### Marcos Peñalver^1^, Patricia Martínez^2^, Magdalena Lluch^2^, Alfonso Malet^2^

#### ^1^Probelte Pharma, Murcia, Spain; ^2^Allergy and Clinical Immunology Service, Hospital de Nens de Barcelona, Barcelona, Spain

##### **Correspondence:** Marcos Peñalver (drpenalver@probeltepharma.es)

OBJECTIVE

The aim has been to evaluate the safety of Beltavac Polimerizado® of mites in young children after administration of a rush schedule and efficacy after nine months of treatment.

METHODS

This is a retrospective observational study with an evaluation of 40 medical histories of children between 3 and 11 years whose were diagnosed with rhinoconjunctivitis and/or bronchial asthma by sensitization to mites. These children had been treated with Beltavac Polimerizado® (mix *Dermatophagoides pteronyssinus* and *Dermatophagoides farinae*) in a rush schedule (0.2 + 0.3 ml with an interval between doses of 30 minutes and 0.5 ml as maintenance dose after one month). The mean age of children was 7.1 years and diagnosis was rhinitis + asthma (50%), rhinitis (25%), asthma (15%), rhinoconjunctivitis + asthma (5%), rhinoconjunctivitis (5%). The variable of study to evaluate safety was the number of adverse reactions per dose classified according to EAACI criteria. The variables used to evaluate efficacy were the clinical status of the patients, medication use and specific IgE and IgG4 values after nine months of treatment. This study was classified as EPA-OD by AEMPS and evaluated and approved by the ethics committee of Hospital Virgen de la Arrixaca in Murcia.

RESULTS

In relation to safety 6 adverse reactions in the 120 doses administered were observed (5%). The classification of adverse reactions was: two immediate local reactions (<5 cm), three delayed local reactions (one <5 cm; two between 5-10 cm) and one systemic reaction (grade 1). All patients tolerated the maximum dose and there was no treatment suspension.

Regarding efficacy, there was a clinical improvement in all patients and reduction of use of medicines in 85% of patients after nine months of treatment. There were no statistically significant differences in total and *D. pteronyssinus*, *D. farinae*, Der p 1 specific IgE, but was a statistically significant reduction of Der p 2 specific IgE. In relation to IgG4 there was a statistically significant increase of specific *D. pteronyssinus* and *D. farinae* IgG4.

CONCLUSIONS

The treatment with Beltavac Polimerizado® of mites administered by a rush schedule is safe in children between 3 and 11 years. The clinical and immunological data showed an efficacy of this treatment.

## A23 Effect of pollen immunotherapy on the oral allergy syndrome

### Young-Hee Nam^1^, Hyun Jung Jin^2^, Soo-Keol Lee^1^

#### ^1^Department of Internal Medicine, College of Medicine, Dong-A University, Busan, Korea; ^2^Department of Internal Medicine, Yeungnam University Medical School, Daegu, Korea

##### **Correspondence:** Young-Hee Nam (dr00nam@hanmail.net)

BACKGROUND

Twenty-three to 70% of individuals with pollen allergy experience enoral symptoms after eating raw fruits and vegetables. The oral allergy syndrome (OAS) reflects a phenomenon based on the cross-reactivity of specific IgE antibodies towards epitopes of certain pollen and food proteins. Improvement of the OAS in pollen-allergic subjects using specific immunotherapy (SIT) has been reported to be approximately 50%. A few studies have reported improvement of OAS mainly focused on apple following birch pollen immunotherapy.

OBJECTIVE

We aimed to investigate the effect of SIT on OAS.

METHODS

Fifteen birch or mugwort pollen-allergic subjects with OAS who received SIT at least 1 year were surveyed by questionnaire. Skin prick test and serum specific IgE to birch or mugwort pollen were determined.

RESULTS

Eight of 15 (53%) showed improvement of OAS. Skin-test reactivity and specific serum IgE to pollen decreased in 70% and 67% of the subjects, respectively. The subjects with improvement of OAS were a higher proportion of female and lower serum total IgE levels than those without improvement. There was no significant difference in skin-test reactivity, specific serum IgE, and SIT duration. The symptom score of OAS was significantly correlated with which of allergic disease.

CONCLUSIONS

SIT with birch or mugwort pollen improved OAS. Female and low serum total IgE may be related with SIT impact on OAS.

## A24 Skin test reactivity to *Dermatophagoides pteronyssinus* and *farinae* among Thai children with respiratory allergy

### Prapasri Kulalert^1^, Paskorn Sritipsukho^2^, Jayanton Pathumanond^3^

#### ^1^Division of Clinical Epidemiology, and Division of Allergy and Immunology, Deptartment of Pediatrics, Faculty of Medicine, Thammasat University (Rungsit Campus), Pathum Thani, Thailand, and Thammasat University Center of Excellence of Asthma, Allergy and Immunology, Thammasat University Hospital, Pathum Thani, Thailand; ^2^Division of Allergy and Immunology, Department of Pediatrics, Faculty of Medicine, Thammasat University (Rungsit Campus), Pathum Thani, Thailand, and Center of Excellence in Applied Epidemiology, Thammasat University (Rungsit campus), Pathum Thani, Thailand; ^3^Division of Clinical Epidemiology, Faculty of Medicine, Thammasat University (Rungsit Campus), Pathum Thani, Thailand

##### **Correspondence:** Prapasri Kulalert (kulalertp@yahoo.com)

BACKGROUND

*Dermatophagoides pteronyssinus* (Der p) and *Dermatophagoides farinae* (Der f) are two common species causing sensitization in children with asthma and allergic rhinitis. *In vitro* studies have demonstrated IgE-binding crossreactive epitopes between two species. Currently, Der p and Der f are both commercially available extracts for skin prick testing. However, there are few studies evaluating the concordance and correlation of skin test reactivity to these allergens in children.

OBJECTIVE

The purpose of this study was to assess agreement of sensitization between Der p and Der f allergen extracts in children. Correlation of skin test responses in wheal diameters between them was evaluated.

METHODS

This study was a retrospective medical chart review of the skin prick test results among children aged 2 to 18 years old who presented with symptoms suggestive of allergic rhinitis and/or asthma at the Pediatric Allergy Clinic of Thammasat Hospital, Pathum Thani, Thailand. All patients underwent skin prick test with Der p and Der f commercial allergen extracts. Sensitization was defined as > 3 millimeters in wheal diameter from skin prick testing. The kappa was used to evaluate the agreement of sensitization and Spearman analysis (rho) was used for assessing the correlation of mean wheal diameter between both extracts. The patients were divided into 3 groups; preschool (2-6 years old), school-aged (7-12 years old) and adolescent (13-18 years old) for analysis skin test reactivity according to the age group.

RESULTS

Of the 280 patients, 181 (64.6%) were male. The mean age was 7.6 ± 3.5 years. 267 (95.4%) patients had allergic rhinitis and 72 (25.7%) had asthma. 157 (56.1%) of patients were positive to Der p and 146 (52.1%) were positive for Der f, respectively. The correlation of mean wheal diameter between Der p and Der f allergen extracts showed strong correlation, all age (rho = 0.9; p < 0.001), preschool (2-6 years old; rho =0.92; p < 0.001), school-aged (7-12 years old; rho = 0.88, p < 0.001) and adolescent (13-18 years old; rho=0.89, p < 0.001). The agreement of sensitization between Der p and Der f was substantial to almost perfect, all age (kappa=0.83), preschool (2-6 years old; kappa=0.87), school-aged (7-12 years old; kappa= 0.83) and adolescent (13-18 years old; kappa = 0.72), respectively.

CONCLUSIONS

This study showed a strong correlation and substantial to almost perfect agreement of sensitization on skin test response between Der p and Der f in children. A trend of agreement was decreased with increasing age when stratified by age group.

## A25 The lymphocyte activation test – Clinical utility in delayed skin reactions in paediatric age group: A prospective study

### Krasimira Baynova, Marina Labella, Teresa De Aramburu, Manuel Prados

#### Allergy Unit, University Hospital Virgen del Rocío, Seville, Spain

##### **Correspondence:** Krasimira Baynova (krasi1024@yahoo.com)

BACKGROUND

Drug hypersensitivity reactions (DHR) are classified as immediate and non-immediate/delayed reactions, depending on their onset during treatment. Non-immediate drug-induced skin reactions in children are not common but they suppose a challenge in our daily practice. Drug provocation test (DPT) cannot be considered in many occasions because of the risk of severe reactions. Safer *in vitro* tests are being sought in order to establish the diagnosis in non-immediate DHR.

OBJECTIVE

Our aim was to evaluate the utility of the lymphocyte activation test (LAT) in the diagnosis of delayed skin DHR in children.

METHODS

We prospectively studied 32 children (from 1 to 18- year-old) who consulted us for suspect delayed mild-moderate skin DHR. The involved drugs were beta lactam antibiotics, non beta lactam antibiotics and nonsteroidal anti-inflammatory drugs (NSAIDs). DHR had occurred 4 weeks-1 year before testing. To all patients we performed patch test, LAT and previous informed consent was signed by the parents - a DPT.

To carry out the LAT, peripheral mononuclear patient’s cells were isolated and incubated with the studied drug during 48 hours. We evaluated the surface molecule CD69 by flow cytometry as an *in vitro* indicator for T-cell lymphocyte activation.

RESULTS

34 LAT were performed (few drugs involved in some patients). 25 of them had a negative result and 24 of the respective DPT were negative too (predictive negative value – 96%). 9 of the performed LAT had a positive result and showed a low positive predictive value (11%) when DPT was carried out, just 1 of the DPT was positive too (mild cutaneous reaction). None of the patch test was positive.

CONCLUSIONS

LAT has an excellent predictive negative value in our group of patients. LAT would be useful in cases of delayed skin DHR when the child was polimeidacted and all involved drugs had to be studied cautiously to find the culprit drug and to reject the involvement of the rest of the drugs. Further studies are needed to confirm our findings.

## A26 Burden of allergic disease and child well-being

### Leena Haanpää^1^, Jasmin Aarnio^3^, Merja Nermes^2^, Piia af Ursin^1^, Anne Kaljonen^3^, Erika Isolauri^2^

#### ^1^Child and Youth Research Institute, Department of Clinical Medicine, University of Turku, Turku, Finland; ^2^Department of Pediatrics and Adolescent Medicine, Turku University Hospital, Turku, Finland; ^3^Faculty of Medicine, Department of Clinical Medicine, University of Turku, Turku, Finland

##### **Correspondence:** Leena Haanpää (lehaan@utu.fi)

BACKGROUND

The prevalence of allergic diseases and the severity of symptoms continue to increase worldwide, and velocity of propagation is highest in children. Above the burden on the society, allergic diseases hold a key position with regard to the burden at individual and family level. However, formal studies on the psychosocial impacts of allergic disorders on subjective wellbeing of children remain obscure. Severity of allergic disease is approximated by the extent of symptoms in daily life and patients’ perception of well-being.

METHODS

The cross-sectional study included 1,947 children from Finland, which is a subsample of the worldwide Children’s Worlds, the International Survey of Children’s Well-Being (ISCWeB). A randomly selected, nationally representative data collection was completed in 2016. Two psychometric wellbeing scales (SLSS, BMSLSS) were applied to determine the connection between allergic diseases and life satisfaction of 10- to 12-year-old children.

RESULTS

Finnish children evaluated their well-being as excellent; more than 80% of 10- and 12-years-olds are very satisfied with their lives. Especially children with atopic dermatitis (24%) were more likely to report reduction both in SLSS life satisfaction than those with no atopic dermatitis (6% vs. 11%, OR = 1.77, 95% CI = 1.23 - 2.56, p = 0.0021) and BMSLSS life satisfaction (14% vs. 20%, OR = 1.52, 95% CI = 1.15 – 2.0, p = 0.0029). In multiple linear regression analysis, life satisfaction, as measured by SLSS (F (8, 1545) = 6.92, p < .0001, R2 = .0296) and BMSLSS (F (8, 1445) = 13.82, p < .0001, R2 = .0659), was associated with younger age, working parents, not having atopic dermatitis, and practicing sports regularly.

CONCLUSIONS

Active allergic disease reduces a child’s own perception of wellbeing. Results highlight the importance of early diagnosis and treatment. More attention should be paid to the psychological status and treatment compliance in children with allergic disease.

## A27 Mapping indoor allergens in the homes of allergic children in urban India

### Nandana Bala^2^, Ketaki Bhagwat^1^, James Hindley^3^, Martin Chapman^4^, Sivasankar Baalasubramanian^1^

#### ^1^Indoor Biotechnologies India Pvt Ltd, Bangalore, India; ^2^Rainbow Children’s Hospital, Bangalore, India; ^3^Indoor Biotechnologies Ltd, Cardiff, Wales, United Kingdom; ^4^Indoor Biotechnologies Inc, Charlottesville, VA, USA

##### **Correspondence:** Nandana Bala (nandanabala@yahoo.com)

BACKGROUND

Children are bearing the brunt of a dramatic increase in incidence of allergic diseases in the developing world, including India. Indoor allergens derived from dust mites, cockroaches, rodents, pets and fungi are associated with asthma and allergic diseases. Allergen avoidance is a crucial component in the management of these conditions. There is little data from India on indoor allergens and exposure levels that trigger symptoms.

OBJECTIVE

The study aims to identify and quantify indoor allergens in homes of children with proven allergic disease in urban India.

METHODS

The study has been designed to cover 20 Indian cities with different climatic conditions. Phase 1 participants were recruited from a pediatric allergy clinic in one of the cities (Bangalore). Children with features of allergic disease testing positive to one or more allergens by skin prick testing (SPT) were included. Dust samples from homes of patients and non-allergic controls were collected using DUSTREAM ® collectors. Analysis for the presence of allergens was done by MARIA (Multiplex Array for Indoor Allergens) assays.

RESULTS

Samples from 10 controls and 25 cases were analysed. The allergens assayed were Der p1, Der f1 MG2, Can f1, Fel d1, Mus m1, Rat n1, Bla g2, Alt A1 and Asp f1. Majority of homes had significant levels (>2mcg/gm dust) of house dust mite (HDM) allergens with over 2/3^rds^ having very high levels (>10mcg/gm dust). Levels of other allergens were relatively low. Allergen levels were similar in homes of both cases and controls.

CONCLUSIONS

This is the first study of its kind in India. Most children with allergic disease in this urban setting were sensitised to HDM Allergens. The majority of the homes had significant levels of HDM allergens. However, high level of environmental exposure alone may not lead to development of allergic disease. The results will guide use of comprehensive environmental intervention measures to achieve reduction in allergen exposure.

Further studies are needed to confirm if quantifying the level of exposure to relevant allergen can guide immunotherapy and also if primary prevention of allergy is possible by reducing exposure to potentially sensitising agents like HDM in the environment.

## A28 Probiotic microorganism *Lactobacillus reuteri* impact on the prevalence of allergic asthma in five years old Slovene children

### Lilijana Besednjak-Kocijančič (bekoli86@gmail.com)

#### Zdravstveni dom Nova Gorica, and Primary Pediatric Centre, Nova Gorica, Slovenia

BACKGROUND

The intestinal microflora is very important for maturation of the host’s innate and adaptive immune responses. Perinatal probiotics supplementation has been shown to be effective in the primary prevention of atopic dermatitis. Effect of probiotics on prevention of allergic asthma is less certain. Many reports suggest that certain probiotic strains have potent immunomodulatory activity in allergic asthma.

OBJECTIVE

The aim of this study was to evaluate the efficacy of probiotic microorganism *Lactobacillus reuteri* (LR) in the prevention of the development and exacerbations of asthma in children.

METHODS

This prospective study included 316 maturely born infants with a positive history of parental allergy confirmed by allergy testing. According to the addition of LR in to the child diet, they were divided in group A-201 infants exclusively breastfed for 4-6 months, and group B-115 infants breastfed with addition of LR from fourth week of life for 12 weeks. Every child was followed up by the same paediatrician until five years old. The prevalence of doctor-diagnosed asthma, frequency and duration of asthma attacks and bronchodilator consumption was observed. Statistical analysis was performed with SPSS 11.0 using chi-square analysis with Yates’ correction. P-values less than 0.05 were considered significant.

RESULTS

At the age of 5 years, the prevalence of asthma tended to be higher in group A (group A: 15.4%; group B: 8.7%), (P=0.08). Higher frequency of asthma attacks (group A: 8.2; group B: 5.8 attacks/asthmatic child) and longer duration of asthma attack (group A: 11.4; group B: 9.3 days/attack) was observed in group A. Consumption of bronchodilators was also greater in group A (Group A: 12.1; group B: 6.3 prescriptions per asthmatic child).

CONCLUSIONS

Our study confirms that addition of LR to early child’s diet has not preventive effect in asthma development, but there is some beneficial effect on the course of asthma in children.

## A29 Immunoproteomic study on allergens of *Aspergillus oryzae*: A major allergy sensitizing mould

### Koyel SenGupta, Swati Gupta Bhattacharya

#### Division of Plant Biology, Bose Institute, Kolkata, India

##### **Correspondence:** Koyel SenGupta (koyelsengupta.bot@gmail.com)

BACKGROUND

Inhalation of fungal aeroallergens is a substantial risk factor for a wide range of allergic diseases.

OBJECTIVE

An aeromycological monitoring was performed in an urban megacity, Kolkata, India, to quantify fungal aerospores and to identify allergenic components of *Aspergillus oryzae*, one of the most dominant fungi, by immunoproteomic analysis.

METHODS

Airborne fungi were biomonitored during two years and fungal spore calendar was prepared. Spore concentrations were correlated with different meteorological parameters and hospitalization data. After skin prick test, allergenic potential of *Aspergillus oryzae* was tested by ELISA and Histamine assay. Both extracellular and intracellular proteins were analyzed by immuno-proteomic approach. After one and two dimensional gel electrophoresis, IgE reactive proteins were detected from 1D and 2D Immunoblot (12% and 15%). For intracellular proteins, presence of glycoprotein in the allergen profile and the role of sugar moiety in IgE-binding were assessed by Periodic Acid Schiff’s staining followed by meta-periodate modification. IgE reactive spots were identified by MALDI-TOF-TOF. Major allergen was purified partially by anion exchange chromatography.

RESULTS

From biomonitoring, *Aspergillus* were found to be the most predominant fungi followed by *Cladosporium*, *Penicillium*, etc. Spore concentrations were greatly influenced by meteorological parameters and were positively associated with hospitalization records. 18 intracellular and 10 extracellular allergens of *Aspergillus oryzae* were detected from 2D immunoblot. For intracellular proteins, three allergens were found having glycol moiety as a major contributor of IgE-epitope by Periodic Acid Schiff’s staining followed by meta-periodate modification. 67% of *Aspergillus oryzae* allergic patients were sensitized to glucan 1, 3-beta-glucosidase A (45.434 kDa) by IgE immunoblot, which was identified as major allergen by MALDI-TOF-TOF. This major allergen (pI 4.63) was partially purified by anion exchange chromatography and showed its IgE reactivity by 1D immunoblot confirming successful partial purification of this allergen.

CONCLUSIONS

In the present study, glucan 1, 3-beta-glucosidase A is identified as major allergen from *Aspergillus oryzae*, which has been reported for the first time. Immuno-proteomic identification of all IgE reactive proteins is helpful for proper diagnosis and immunotherapy of atopic diseases.

## A30 High healthcare resource use in children with severe or poorly controlled asthma

### Bradley E Chipps^1^, Evgeniya Antonova^2^, Amanda M Kong^3^, Ahmar Iqbal^2^, W Gerald Teague^4^

#### ^1^Capital Allergy and Respiratory Disease Center, Sacramento, CA, USA; ^2^Genentech, Inc., South San Francisco, CA, USA; ^3^Truven Health Analytics, an IBM Company, Cambridge, MA, USA; ^4^Child Health Research Center, University of Virginia School of Medicine, Charlottesville, VA, USA

##### **Correspondence:** Ahmar Iqbal (iqbal.ahmar@gene.com)

OBJECTIVE

We report real-world healthcare use in privately- or publicly-insured children with severe or poorly-controlled asthma vs those without asthma in the United States.

METHODS

In US claims databases (years 2008-2014), we identified privately- and publicly (Medicaid)-insured children (6-11 years old) with severe or poorly-controlled asthma (≥2 medical claims for asthma, ≥2 filled pharmacy claims for any asthma medication per 12 months, and ≥1 filled pharmacy claim for high-dose inhaled corticosteroids or chronic oral corticosteroids or ≥5 pharmacy claims for short-acting beta-agonists in 3 consecutive months). We matched (1:1) children with vs without asthma by demographic characteristic and evaluated general healthcare use rates per patient-per-year.

RESULTS

We matched children with asthma vs without asthma (private: n=33,108 in the asthma group and the same number in the non-asthma group; Medicaid: n=29,493 in the asthma group and the same number in the non-asthma group), followed for a mean of 3.2 (private) and 3.6 years (Medicaid). Privately-insured children with asthma vs without asthma had the following mean (95% CI) rates of healthcare use: inpatient admissions (0.09 [0.09-0.10] vs 0.02 [0.01-0.02]), hospital days (0.35 [0.33-0.38] vs 0.08 0.07-0.10]), ED visits (0.54 [0.53-0.55] vs 0.22 0.21-0.22]), office visits (7.51 [7.46-7.57] vs 3.27 [3.24-3.29]). Medicaid-insured children with asthma vs without asthma had the following mean (95% CI): inpatient admissions (0.13 [0.12-0.13] vs 0.03 [0.03-0.03]), hospital days (0.57 [0.53-0.61] vs 0.32 [0.27-0.37]), ED visits (1.03 [1.02-1.05] vs 0.41 [0.41-0.42]), office visits (7.30 [7.23-7.36] vs 2.85 [2.82-2.88]. All p-values <0.01.

CONCLUSIONS

Children with severe or poorly-controlled asthma use more (>2-fold) healthcare services than children without asthma. The results are consistent for privately- and publicly-insured children.

## A31 Omalizumab treatment increases the likelihood of being free of exacerbations and healthcare use in children

### Bradley E Chipps^1^, Evgeniya Antonova^2^, Benjamin Trzaskoma^2^, Benjamin Ortiz^3^, Brandee Paknis^3^, Amar Iqbal^2^, Karin Rosen^2^ Stanley Szefler^4^

#### ^1^Capital Allergy and Respiratory Disease Center, Sacramento, CA, USA; ^2^Genentech, Inc., South San Francisco, CA, USA; ^3^Novartis Pharmaceuticals Corporation, East Hanover, NJ, USA; ^4^Pediatric Asthma Research Program, Children’s Hospital and University of Colorado School of Medicine, Colorado, Aurora, CO, USA

##### **Correspondence:** Amar Iqbal (iqbal.ahmar@gene.com)

BACKGROUND

Omalizumab has been reported to reduce exacerbations in school-age children with moderate or severe persistent asthma.

OBJECTIVE

We aimed to describe the effect of omalizumab treatment on the likelihood of remaining free of asthma exacerbations and healthcare resource use.

METHODS

This randomized, double-blind, placebo-controlled, phase III clinical trial (years 2004-2008) enrolled children ≥6 to <12 years old with allergic asthma and randomized them (2:1) to receive omalizumab or placebo. Background inhaled corticosteroid (ICS) dose remained constant during weeks 1-24; investigators could reduce ICS doses during weeks 25-52. This post-hoc analysis compared the percentages of children (omalizumab vs. placebo) free of severe exacerbations (those that required systemic corticosteroids and had a peak expiratory flow or FEV1 <60% of personal best), clinically significant exacerbations (worsening symptoms requiring doubling of baseline ICS dose or systemic corticosteroids), clinician reported exacerbations, and healthcare resource use (hospitalizations and outpatient services) during weeks 1-52. P-values were estimated via a chi square test.

RESULTS

Among patients treated with omalizumab (n=384) or placebo (n=192), the majority (n=365) had severe persistent asthma. During weeks 1-52, more omalizumab- than placebo-treated children were free of: severe exacerbations (88.0% vs. 79.2%, 95% CI: 2.2-15.4%, p=0.005), clinically-significant exacerbations (52.9% vs 39.6%, 95% CI: 4.8-21.8%, p=0.003), and any clinician-reported exacerbation (25.3% vs. 13.0%, 95% CI: 5.9-18.7%, p <0.001).

A higher percentage of omalizumab- than placebo-treated children remained free of hospital admissions (95.6% vs 90.6%, p=0.024) and unscheduled doctor visits (71.6% vs 62.5%, p=0.032). The percentages of omalizumab- vs placebo-treated children free of ED visits were: 94.3% vs 91.7% (p=0.246).

CONCLUSIONS

Omalizumab treatment helps children with moderate-to-severe asthma be free of severe exacerbations, clinically-significant exacerbations, any clinician-reported exacerbations, hospital admissions and unscheduled doctor visits.

## A32 Forced oscillation measurements for the assessment of respiratory function in Emirati children

### Afaf Alblooshi, Suleiman Al-Hammadi

#### Deptartment of Pediatrics, UAE University, Al-Ain, United Arab Emirates

##### **Correspondence:** Suleiman Al-Hammadi (suleiman.alhammadi@uaeu.ac.ae)

BACKGROUND

Forced oscillation technique (FOT) facilitates the quantification of respiratory function in young children. Outcomes from FOT and their utility in non-Caucasian populations are not well understood.

OBJECTIVE

This community-based study explored the use of the FOT in school-aged children with and without respiratory problems in the United Arab Emirates (UAE).

METHODS

Three hundred seventy-one Emirati children were recruited from primary schools and the pediatric respiratory clinic at Tawam Hospital. Forced oscillation measurements were successful in 351 (95%) children, aged three to 12 years. Standardized respiratory history was available in 330 children (109 with and 221 without respiratory problems) using a modified global asthma network questionnaire.

RESULTS

Logistic regression model that included weight, height, age and gender, showed only height had significant prediction of FOT values (p<0.001). Prediction equations for resistance (Rrs), reactance (Xrs) and area under the curve (AX) were generated based on height, e.g., Rrs at 5 hertz (Rrs5) = 20.67 + (-0.10 × height), R2=0.38. Using the prediction equation derived from the children without respiratory symptoms Z-scores were calculated. There was statistically significant difference in Z-score at Rrs5 between children with and without respiratory symptoms (p<0.05, independent sample t-test).

CONCLUSIONS

FOT measurements were feasible in Emirati school-children. The procedure was brief and well perceived by the children, families, and school staff. The results identified some children with respiratory symptoms and high airway resistance.

## A33 Use of an electronic device and a computerized mathematic algorithm to detect the allergic food reactions through the analysis of heart rate variability

### Arantza Vega^1^, Raquel Gutiérrez-Rivas^2^, Ana Maria Alonso^1^, Juan Maria Beitia^1^, Maria Belén Mateo^1^, Remedios Cárdenas^1^, Juan Jesús García-Domínguez^2^

#### ^1^Allergy Service, Hospital Universitario de Guadalajara, Guadalajara, Spain; ^2^Electronic Department, Polytechnic School, University of Alcalá, Madrid, Spain

##### **Correspondence:** Arantza Vega (arantza.vega@gmail.com)

BACKGROUND

Challenge test (CT) is the gold-standard for the diagnosis with food allergy. Its implementation involves a high consumption of time and resources and implies the risk of severe adverse reactions appareance. Currently, there is not a diagnostic tool able to perform an early detection of the allergic reactions, thus reducing the length of the challenge test and decreasing the onset of severe allergic reactions. Heart rate variability has been used for help to the diagnosis of several illnesses. It has been recently used for the detection of allergic reactions. We explore the use of a heart rate variability monitoring device to detect in real time the allergic reactions in patients undergoing a CT.

METHODS

Patients with suspected food allergy that, following the normal diagnostic protocol, were undergoing a CT and whose parents or tutors agreed to participate. A remote monitoring device called Shimmer was placed from10 minutes before the start of the test until Its completion. Increasing doses of the food were administered at 30 minutes interval untill the end of the test or until the onset of allergic symptoms. Heart rate variability was analysed by the proposed algorithm, and Its results were compared to the CT results.

RESULTS

Sixty six patients (52 children and 14 adults) were studied: 30 females, 36 males, mean age 5,6 years (IQR 2-11 y). Foods tested included milk (20), egg (18), fish (13), fruits (6) and others (9). The food CT was positive in fourteen of them (21%). The algorithm detection showed a positive result in 41 patients and a negative one in 25. Its sensitivity and specificity were 93% and 46% respectively. The global negative predictive value was 96%. The detection was a mean of 64 minutes before the onset of symptoms.

CONCLUSIONS

The use of a heart rate variability device with a mathematic algorithm can be a useful tool to increase safety in food allergy diagnosis. Further studies are needed.

## A34 Children and adolescents out-of-hospital asthma deaths in Brazil

### Raquel Pitchon dos Reis^1,2^, Cristina Gonçalves Alvim^1^, Claudia Andrade^1^, Adriana Reis^1^, Henrique Ribeiro^1^

#### ^1^Department of Pediatrics, Federal University of Minas Gerais, Belo Horizonte, Brazil; ^2^Allergy Department, Mater Dei Hospital, Belo Horizonte, Brazil

##### **Correspondence:** Raquel Pitchon dos Reis (pitchonreis@ig.com.br)

BACKGROUND

Asthma is a common chronic respiratory disease in childhood and adolescence and it is associated with reduced quality of life, hospitalizations and rarely deaths.

OBJECTIVE

The objective of the study was to evaluate out-of-hospital asthma deaths in Brazil, from 1996 to 2014, occurring in the age group between zero and nineteen years old.

METHODS

This is an ecological study, based on the database collected in DATASUS, CGIAE (General Coordination of Information and Analysis Epidemiologic). The identification of deaths was conducted by the ICD (International Classification of Diseases and Related Health Problems), 10th revision, J-45 (asthma) and J-46 (status asthmaticus) as root causes in Brazil. The variable studied was the place of death, considering wether it occurred in a hospital or out-of-hospital (another health establishment, residence, public road, others, ignored).

RESULTS

From January 1996 to December 2014, there were 4872 asthma deaths in Brazil, in children and adolescents between zero and 19 years. Of these deaths 3815 (78,5%) occurred in hospital e 21,5% out-of-hospital. Considering the age, in the group under one year, 17,3%, between 1 to 4 years: 20,9%, from 5 to 9 years: 22,3%, in group 10 to 19 years, 27,2%, ocurred out-of-hospital (another health establishment, residence, public road, others, ignored). The difference was statistically significant in the group of 10 to 19 years (p < 0.005).

CONCLUSIONS

It would be desirable for all cases of asthma attacks to occur in a hospital environment and with broad access to tertiary resources. Out-of-hospital asthma deaths were significantly higher among adolescents. Most asthma deaths are avoidable. Factors related to access to health services for asthma care, availability of medications and adherence to treatment, association with others clinical diseases, and sociodemographic issues may increase the risk of death from the disease. There are well-established strategies for reducing morbidity and mortality from asthma, that includes the regular use of preventive medications, especially inhaled corticosteroids, education of the patient and his family and portability of a plan of action for the early treatment of exacerbations. The main objective of studies about asthma mortality is the orientation and the elaboration of public health policies, strategies and priorities for prevention and treatment of the disease.

## A35 Seasonal ragweed pollen exposure during infancy is associated with increased risk of subsequent childhood rhinoconjunctivitis

### Carmen Panaitescu Bunu^1^, Laura Marusciac^2^, Sorin Paralescu^1^, Paul Tamas^2^

#### ^1^Victor Babes University of Medicine and Pharmacy, Timisoara, Romania; ^2^OncoGen Research Center, Emergency Clinical County Hospital, Timisoara, Romania

##### **Correspondence:** Carmen Panaitescu Bunu (cbunu@umft.ro)

BACKGROUND

Ragweed pollen (*Ambrosia artemisiifolia*) represents approximately 20% out of the total pollen concentration in the Western part of Romania, and is highly allergenic, inducing severe allergic symptoms. Few studies have focused on pollen exposure and allergic manifestations in children.

OBJECTIVE

The study aims to evaluate the association between higher ambient levels of ragweed pollen in the first 2-4 years of life and risk of sensitization and development of allergic rhinitis in children at age of 12, in comparison with adult subjects.

METHODS

Using the patient cohort with allergic respiratory symptoms (n = 10,883) evaluated between 2008 and 2016 in the Allergy Outpatient Clinic Timisoara, we examined allergic sensitization (SPT > 3 mm to at least one of birch, rye grass, ragweed, house dust-mite, dog and cat dander) by age 2 and the development of allergic rhino-conjunctivitis or/and asthma at age 12, in comparison with adult subjects.

RESULTS

Most of adult patients (63.5%) were polysensitized to both indoor and outdoor allergens, out of which, 60% were sensitized to ragweed. In monosensitized adult patients, ragweed allergy was present in 42%, more than to any other type of airborne allergen, followed by house dust mite (38.5%). All patients with a disease history longer than 10 years had developed allergic asthma. For the period 2015-2016 there were 6 cases of children between 1.5 and 4 years of age, and a total of 57 children under 12 years of age with documented ragweed allergic rhinoconjunctivitis.

CONCLUSIONS

Exposure to high concentrations of ragweed pollen in infancy appears to increase the risk of rhinoconjunctivitis in children under 4 years. These results support the hypothesis that high level of ragweed pollen can induce sensitization and the development of allergic symptoms early in life, and progression to asthma in adulthood, even if the period of exposure is less than 3 months per year.

## A36 Allergy unit in oncology institute – First national experience about the first 600 desensitizations to chemotherapeutics and monoclonal antibodies

### Enrique Martí Guadaño^1^, Carolina Escobar Bolaños^1^, Natalia Martí José^1^, Pol Pau Casanovas^2^, Gemma Biarnés Rib^2^, Mariana Castells^3^

#### ^1^Allergy Unit, Institut Català d’Oncològia, Barcelona, Spain; ^2^UDAM Allergy Unit, Barcelona, Spain; ^3^Desensitization Unit, Brigham and Women’s Hospital, Boston, MA, USA

##### **Correspondence:** Enrique Martí Guadaño (udam93@hotmail.com)

BACKGROUND

There is an increase in malignant proliferative diseases as well as in allergic pathology. The Institut Català d’Oncologia, (ICO), covers 4 million inhabitants in the Barcelona area, publishes 4 per thousand of adverse reactions to chemotherapy, mediated or not by allergic mechanisms, and, in most cases, leaving the first therapeutic lines, for second one, safer, but less effective and undoubtedly higher direct costs.

OBJECTIVE

Our aim is to reverse that process, to get the opportunity to keep those patients with the best of possible medications.

METHODS

Two part-time allergists, one nurse specialized in drug allergy, one pharmaceutical, under the coordination of one Head of Unit. First visit, skin tests, risk stratification and desensitization decision, place and number of steps. The desensitization protocols used are from Brigham and Women’s Hospital of Boston, world pioneers of this technique. In cases of new drugs, this hospital and its Desensitization Unit led by Professor Mariana Castells was consulted for her experience.

RESULTS

185 patients, with different types and stages of cancer, predominantly ovary, breast and colon; 601 cycles of desensitization, mainly to Platins and taxanes; 98% of successes (reaching final dose prescribed by oncologist) 70% were 12-step cycles and 25% of 16; All in Day Hospital, except 2 in Intensive Care.

CONCLUSIONS

The results show that a Chemotherapy Drug Desensitization Unit, managed by allergists, increases the population’s survival expectations with cancer and allergy to necessary drugs, being able to maintain the first therapeutic line, recommended by the international guidelines, and that of another form, they are confronted with second choices, safer perhaps, but clearly less effective.

## A37 A case report of lethal food-induced anaphylaxis (FIA) in an adolescent

### Talía de Vicente Jiménez (taliadevicente@hotmail.com)

#### Pediatrics & Allergy Department, Hospital Central de la Defensa Gómez-Ulla, Madrid, Spain

BACKGROUND

Food can be a highly significant trigger for IgE mediated anaphylaxis in children. Bearing in mind the limitation of epidemiologic challenges in determining the burden of food-induced anaphylaxis (FIA), it is estimated during recent years that the incidence of FIA in children (likely affects 8%) is increasing as well as in adults (nearly 5%), with growing evidence of an increase in prevalence: Hospitals admissions for FIA have more than doubled in the last decade.

METHODS

15 year-old-girl feeling stomach pain, facial swelling, onset of acute bronchospasm, reduced blood pressure and syncope, within an hour at school, after having for breakfast milk with a new brand of a nut-cereal bar at home. Her teachers performed 15 minutes of insufflation without cardiac massage until the emergency services arrived. They performed 8 minutes of basic CPR, 10 minutes of advanced CPR: Orotracheal intubation, intratracheal and intravenous Adrenaline, intratracheal Salbutamol and Magnesium sulfate. She recovered sinus rhythm. (Total arrest time: 33 minutes). On arrival at pediatric ICU: pH 6.9, pCO2 120mmHg, lactic acid 33mmol, 98% O2 + FiO2 100%, heart rate 118, T 32.4 C, blood pressure 89/ 32mmHg.

RESULTS

History of atopic disease, uncontrolled asthma, food allergy to shellfish, egg, peanut, nut and grass allergy was known, but the patient did not check-up with the allergy specialist. Outcome: Mechanical ventilation was needed, the patient remained in unreactive coma with tendency to hypotension, managed with dopamine. Further studies confirmed allergy history, respiratory deterioration, shock and irreversible end of brain activity after 6 days in intensive pediatric care unit at hospital, where she finally died. Organ donor.

CONCLUSIONS

Intramuscular epinephrine injection is a safe and effective treatment of anaphylaxis in children. A study of anaphylaxis fatalities found that 23% of those who died did not receive epinephrine until cardiac arrest.

Children with systemic allergic reactions should carry epinephrine auto injectors at all times, specially those who have shown severe outcomes and should certainly have one with them at school. In order for epinephrine auto injectors to be effective, children, their families and caregivers must be carefully counselled and educated on how to properly use the devices and when to use them. Instructions on allergen avoidance are key in managing allergies.

CONSENT TO PUBLISH

Consent to publish was obtained from the parents of the patients in this study.

## A38 Allergic sensitization in eosinophilic oesophagitis

### Maurizio Mennini^1^, Carla Riccardi^1^, Papola De Angelis^2^, Francesca Rea^2^, Monica Malamisura^2^, Renato Tambucci^2^, Alessandro Fiocchi^1^, Luigi Dall’Oglio^2^

#### ^1^Division of Allergy, University Department of Pediatrics, Pediatric Hospital Bambino Gesù, Rome, Vatican City, Italy; ^2^Digestive Surgery and Endoscopy Unit, Pediatric Hospital Bambino Gesù, Rome, Vatican City, Italy

##### **Correspondence:** Maurizio Mennini (maurizio.mennini@opbg.net)

BACKGROUND

Eosinophilic oesophagitis (EoE) is a disorder characterized by oesophageal dysfunction and, histologically, by eosinophilic inflammation. Although restrictive diets are often effective, choosing the foods to be eliminated is difficult. Sensitization tests with molecular analysis could allow better-targeted dietary restrictions.

OBJECTIVE

This is a retrospective analysis of patients diagnosed with EoE at our “Pediatric Digestive Surgery-Allergy joint putpatient clinic” over the period 2015-2016, in order to describe their allergenic sensitization.

METHODS

All children who received a histological diagnosis of EoE over the period 1st January 2015 - 30th November 2016 were tested by a standard series of Skin Prick Test (SPT) for aeroallergens and food allergens in accordance with the EAACI guidelines. Specific IgE were performed when clinically indicated. The available data on specific IgE to LTPs from peach (Pru p 3), hazelnut (Cor a 8), peanut (Ara h9), apple (Mal d3) were also analysed. Levels of IgE>0.35 kU/L were considered positive.

RESULTS

Forty-five patients (24 Male; median age 8.53 years; range 1.78-17.12 years; 37 /45 not responder to proton pump inhibitor) were retrospectively analyzed. Four of 37 (10.8%) resulted completely negative for food or aeroallergens.

Twenty subjects (54.1%) showed at least one positivity at SPT for aeroallergens and 13 (35.1%) at least one positivityto foods. Twenty-one out of 37 patients (56.7%) performed specific IgE evaluation: 19/21 returned positive for any aeroallergen and 17/21 for any food. Combining the results of SPT and specific IgE, 12/37 and 15/37 patients were negative for aeroallergens and foods respectively; 9 and 12 were positive for 0-2 aeroallergens or foods respectively and 16 and 10 subjects were positive for more than 2 aeroallergens or foods respectively. Fifteen patients perform LTPs analysis: 10/15 (66.7%) resulted negative for all tested LTPs, while 3 subjects showed positivity only for Pru p3 and 2 patients for Pru p3, Cor a 8 and Ara h9.

CONCLUSIONS

In our EoE caseload, the frequency of sensitization to respiratory allergens is similar to that of food allergens. LTP-syndrome was found in 30% of the tested patients. These data suggest that all EoE patients should be evaluated from an allergological point of view. In this context, many efforts of cooperation between paediatric gastroenterologist and allergist should be provided.

## A39 *Bifidobacterium longum* and *breve* modulation of infant microbiota in food allergy

### Maurizio Mennini^1^, Federica Del Chierico^2^, Tania Napolitano^1^, Silvia Reddel^2^, Pamela Vernocchi^2^, Angelo D’Ambrosio^2^, Lorenza Putignani^2^, Maria Cristina Artesani^2^

#### ^1^Division of Allergy, University Department of Pediatrics, Pediatric Hospital Bambino Gesù, Rome, Vatican City, Italy; ^2^Human Microbiome Unit, Bambino Gesù Children Hospital, Rome, Italy

##### **Correspondence:** Maurizio Mennini (maurizio.mennini@opbg.net)

BACKGROUND

Probiotic’s ability to alter the intestinal microbiotic niches is controversial.

OBJECTIVE

We aim to investigate the ability of a probiotic preparation to modulate the composition of the intestinal microbiota of food-allergic infants.

METHODS

Infants aged 10-14 months with clinical history and IgE sensitization to egg and/or milk underwent diagnostic DBPCFC. Those confirmed allergic (group I) were randomly assigned to receive *Bifidobacterium longum* BB534 (BL), *Bifidobacterium breve* M-16V (BB) and *Bifidobacterium infantis* M-63 (Tribif^®^, Valeas, Milano) for 30 days. Faecal samples at 0, 7, 15, 30, 60 and 90 days from the start of administration of Tribif^®^ were evaluated, after DNA extraction, by quantitative real-time PCR (qPCR). We used specific BB534 (BL) and M-16V (BB) primers obtained after amplification of fragments of 118 and 109 bp. Two control groups of egg and milk-IgE-positive infants with negative food challenge (group II) and IgE-negative healthy infants (group III) were evaluated for BL and BB in basal conditions.

RESULTS

Of 25 infants (15M, 10F, mean age 13.28±2.13 months), 9 (6M, 3F) were DBPCFC-positive to egg (5) or milk (4). Group II was composed by 6 infants (3M, 3F), group III by 6M and 4F. BL was found in the microflora of all children irrespective of their atopic status; BB was absent in 5/25, all in group I. The peak concentrations of BL were reached at 7 days in 5/9, 15 days in 3/9, and 30 days in 1/9. After discontinuation, BL concentration felt to basal levels. When two of these children had to take antibiotics for intercurrent pathologies, the BL concentration dramatically falls. For BB, the peak was reached at 7 days in 2/9, 15 days in 3/9, 30 days in 3/9. BB never colonized the patient who did not show peaks. The two patients who received an antibiotic in the first and second week of administration of Tribif^®^ for intercurrent diseases had BB peaks at 30 days.

CONCLUSIONS

BL and BB are part of the normal bacterial microflora, and the absence of BB colonization may be associated with atopic status. Despite the considerable individual variability in kinetics of the *Bifidobacteria*, the detection of a significant concentration between 0 and 30 days in 8/9 cases with for BL and in 6/9 with BB suggests that the probiotic administered do reach and colonize the intestinal tract. As the effect seems higher in cases in which the baseline concentration is low, Tribif^®^ seems able to restore the ecological niche of *Bifidobacterium* in children where it is depleted.

## A40 Hypoallergencity and tolerance of a new amino acid-based formula in children with cow’s milk allergy

### Lamia Dahdah^1^, Vincenzo Fierro^1^, Claudia Banzato^2^, Luís Angel Echeverría Zudaire^3^, Ana María Plaza^4^, Montserrat Bosque García^5^, Marcel Íbero^6^, Oscar Mazzina^1^

#### ^1^Division of Allergy, University Department of Pediatrics, Pediatric Hospital Bambino Gesù, Rome, Vatican City Italy; ^2^Department of Life and Reproduction Sciences, Section of Pediatrics, University of Verona, Verona, Italy; ^3^Pediatric Allergy Unit, Severo Ochoa Universitary Hospital, Leganés, Spain; ^4^Pediatric Allergy and Clinical Immunology Department, Hospital Sant Joan de Déu, University of Barcelona, Barcelona, Spain; ^5^Division of Pneumology, Hospital Parc Taulí, Autonomous University of Barcelona, Barcelona, Spain; ^6^Allergy Unit, Hospital de Terrassa, Barcelona, Spain

##### **Correspondence:** Vincenzo Fierro (vincenzo.fierro@opbg.net)

BACKGROUND

According to the American Academy of Pediatrics (AAP), a hypoallergenic formula must demonstrate with 95% confidence that 90% of infants with confirmed cow’s milk allergy (CMA) will not react with defined symptoms under clinical trial conditions.

OBJECTIVE

A prospective, double-blind placebo controlled, multi-country study was conducted to assess the hypoallergenicity of a new amino acid-based formula (AAF) in children with cow’s milk allergy (CMA). A commercially available hypoallergenic infant formula was used as a control. Primary aim was the incidence of immediate and/or delayed allergic reactions to double blind placebo controlled food challenge (DBPCFC). Secondary aim, the short-term effects on growth and tolerability of the new AAF were assessed.

METHODS

41 subjects were enrolled and 30 subjects completed the study as per protocol. Patients were recruited after being clinical symptoms-free for one week (or with controlled stable symptoms) and not showing allergic symptoms after the DBPCFC with the new AAF. Subjects consumed at least 250 ml per day during two weeks of study period. Data on anthropometrics, allergic reactions, immunology and health status in general (Symptoms-based clinical score of Vandenplas, SBS) were collected and parents recorded product intake and observed subjective symptoms on the diary.

RESULTS

There were no serious or non-serious adverse events related to any of the two AAF reported in this study. Statistically significant positive difference in weight (p= 0.0019) and height (p < 0.001) were found after 2 weeks of follow-up. All secondary outcomes in gastro-intestinal, respiratory and dermatological tolerability on SBS decreased but without a statistical difference.

CONCLUSIONS

The new formula meets the AAP criteria for hypoallergenicity and the results suggest good tolerability. Patients’ growth seems not hindered by the new formula.

## A41 Tolerance to milk- and egg-free biscuits in children affected by milk and egg allergy

### Vincenzo Fierro^1^, Valeria Marzano^2^, Carla Riccardi^1^, Oscar Mazzina^1^, Lamia Dahdah^1^

#### ^1^Division of Allergy, University Department of Pediatrics, Pediatric Hospital Bambino Gesù, Rome, Vatican City, Italy; ^2^Human Microbiome Unit, Bambino Gesù Children Hospital, Rome, Italy

##### **Correspondence:** Vincenzo Fierro (vincenzo.fierro@opbg.net)

BACKGROUND

A fraction of food-allergic patients are sensitive to minute amounts of foods. They must correctly read the food labels, reporting allergenic ingredients according to local legislation worldwide. In response to these regulations, some industries have also spontaneously added some precautionary allergen labelling (PAL), indicating the possible contamination of foods by allergenic traces.

OBJECTIVE

To evaluate the tolerance to milk and egg-free biscuits among children with severe milk and egg allergy; to explore the possibility that proteomic methods can be used to avoid PAL.

METHODS

A prospective, double-blind placebo, mono-centric controlled study was conducted to assess the hypoallergenicity of “Magretti-Galbusera Frolini con orzo e mais” (MGF) in children with cow’s milk and hen’s egg anaphylaxis. The primary outcome was assessed by double blind placebo controlled food challenge (DBPCFC) with MGF. The presence of milk and/or egg allergen traces in MGF was assessed by nanoLC-MS/MS mass spectrometry. The binding of patients’ serum with MGF proteins was assessed at Western Blot.

RESULTS

We preliminarily report on 25 enrolled children (target per protocol: 29). None of them reacted to MGFs. Patient’s sera (17 tested until now) showed no reactivity with proteins extracted from MGFs. The initial proteomic analysis was negative for egg and milk proteins in MGFs. Unique signature peptides specific to cow’s milk and hen’s egg are being analyzed, and a quantitative analytical method on latest generation triple quadrupole mass spectrometer (QTrap 6550+, Sciex) is being developed to assess allergens at very low concentration (below 10 ppm).

CONCLUSIONS

MGFs seem to meet the AAP criteria for hypoallergenicity. Proteomic techniques may be useful to evaluate the exact amount of protein within the food. Should their sensitivity be pushed up to dose amount of proteins lower than those that can theoretically determine an allergic reaction, they could help in avoiding unnecessary PAL.

## A42 Omalizumab gets tolerance in patients with severe food allergy

### Maurizio Mennini, Maria Cristina Artesani, Oscar Mazzina, Valeria Pecora, Pierluigi Koch, Rocco Luigi Valluzzi, Vincenzo Fierro, Alessandro Fiocchi

#### Division of Allergy, University Department of Pediatrics, Pediatric Hospital Bambino Gesù, Rome, Vatican City Italy

##### **Correspondence:** Maria Cristina Artesani (cristartes@yahoo.com)

BACKGROUND

Therapies for food allergy include elimination diets, Oral ImmunoTherapy (OIT), and the administration of biologics. Omalizumab has been licensed for use in severe allergic asthma and chronic urticaria and its dosing was based on body weight and baseline serum IgE concentration. Omalizumab is currently evaluated as support for OIT.

OBJECTIVE

To evaluate the effect of Omalizumab on food allergy in children with severe asthma.

METHODS

Children with severe asthma and anaphylactic reactions to two or more foods were prospectively enrolled between August 2014 and August 2016. All patients were required to have a baseline IgE between 30 and 1500 IU/ml and body weight not more than 150 kg. Hypersensitivity was confirmed and the threshold dose for each food established by a double-blind, placebo-controlled oral food challenge (DBPCFC) at screening. Patients were assigned to receive either Omalizumab (150, 300, or 450 mg) every two-four weeks. The patients underwent a further oral food challenge to previously positive foods within two to four weeks after the fourth dose.

RESULTS

We enrolled 6 patients (median age 6.98 years; age range 6.98-20.8; 5 males). The mean serum total IgE value was 665 ku/L, and their asthma was out of control despite treatment as per GINA III or IV step. At enrollment, five patients showed allergy for cow milk, four for egg and three for wheat. After treatment, 5/6 patients developed full tolerance to at least one specific food. One patient showed tolerance to baked egg after 5 doses of Omalizumab and to egg after 16 doses; one child developed tolerance to cow milk after 8 doses; one patient developed tolerance to baked cow milk after 8 doses and other two patients showed tolerance to wheat after 4 and 12 doses respectively. The only patient who did not reach full tolerance showed an 85% increase of tolerance to milk (92 mL vs. 14) and an 82% increase of tolerance to egg (25 g vs. 4.4) at DBPCFC. Omalizumab was well tolerated in all children.

CONCLUSIONS

A trial of Omalizumab significantly and substantially increased the threshold of sensitivity to foods on oral food challenge. This effect should translate into protection against most unintended ingestions of food allergens.

## A43 Food protein induced enterocolitis syndrome in patients with Down syndrome

### Valentina Pecora^1^, Diletta Valentini^2^, Maurizio Mennini^1^, Lamia Dahdah^1^, Oscar Mazzina^1^, Francesca Santamaria^1^, Rocco Valluzzi^1^

#### ^1^Division of Allergy, University Department of Pediatrics, Pediatric Hospital Bambino Gesù, Rome, Vatican City Italy; ^2^Division of Infectious disease, University Department of Pediatrics, Pediatric Hospital Bambino Gesù, Rome, Vatican City Italy

##### **Correspondence:** Valentina Pecora (valentina.pecora@opbg.net)

BACKGROUND

Food Protein Enterocolitis Syndrome (FPIES) is a non-IgE food allergy with an unknown prevalence and pathophysiology. Acute FPIES manifests within 1-4 hours after ingestion of incriminated food with repetitive emesis, pallor, lethargy, and water or bloody diarrhea progressing to dehydration and a possible hypovolemic shock.

METHODS

A retrospective descriptive single-center study was conducted. Subjects included in this study were children with acute FPIES to cow’s milk who entered in our institutional follow-up protocol between January 2013 and February 2017.

RESULTS

Among the 21 patients (twelve boys and nine girls), five (23,8%) were affected by Down syndrome. All patients experienced acute FPIES reaction after the ingestion of cow’s milk and four with beef meet (one had a Down phenotype and showed a mild reaction). The average time from ingestion to symptom onset was 120 minutes. The clinical manifestations ranged from mild-moderate (repetitive vomiting with less than three episodes of emesis and pallor) to severe (repetitive vomiting with more than three episodes of emesis, pallor, lethargy, dehydration) or very severe (severe symptoms plus cyanotic appearance and water/bloody diarrhea). Patients with Down syndrome experienced acute FPIES reactions with a severity degree comparable to that reported in other patients. Twelve children were hospitalized, five of them several times. The delay between first reaction and diagnosis was 6.5 months on average. Allergy tests (skin prick test and serum milk protein fractions-specific IgE) were negative in all cases. One patient changed from an acute FPIES to an IgE-mediated phenotype over time.

CONCLUSIONS

To our knowledge, this is the first time that the presence of FPIES reactions in patients with Down syndrome is reported. It is interesting to note that patients with Down phenotype show peculiarly FPIES reactions to cow’s milk proteins accounting for nearly a quarter of the present series.

## A44 Antioxidant supplements for the treatment of allergic rhinitis: A systematic review and meta-analysis of randomized controlled trials

### Anwesha Mukherjee, Amit Kandhare, Subhash Bodhankar

#### Department of Pharmacology, Poona College of Pharmacy, Bharati Videyapeeth Deemed University, Pune, India

##### **Correspondence:** Anwesha Mukherjee (anweshamukherjee007@gmail.com)

*Note: This abstract was presented at the WAO International Scientific Conference (WISC) 2016, Jerusalem, Israel, in December 2016.*

BACKGROUND

Allergic rhinitis is a common inflammatory condition of the upper airways. Antioxidant supplements have proven beneficial in a number of immune-mediated and allergic diseases.

PURPOSE

The current systematic review seeks to synthesize the results of available randomized trials.

METHODS

Study Design: Systematic review and meta-analysis. MEDLINE, EMBASE, Cochrane Library, Clinical-Trials.gov, and Scopus databases were reviewed through January 2016 for randomized controlled trials on defined inclusion criteria.

Study selection: Randomized, controlled trials of various antioxidant supplements that used for the treatment of allergic rhinitis with enough data for meta-analysis.

Data extraction: Two reviewers independently extracted data and assessed study quality. The effect of antioxidant supplements on Rhinitis Quality of Life (RQLQ) scores, Rhinitis Total Symptom Scores (RTSS), as well as total and antigen-specific serum IgE levels were evaluated by meta-analysis. The risk of bias and overall strength of evidence of studies were assessed.

RESULTS

A total of 68 studies were identified, including 8 double-blind randomized controlled trials studies. Various antioxidant supplements, study populations, and outcome measures were utilized in individual trials. Four studies showed a significant clinical benefit from the use of antioxidant supplements in at least one outcome measure when compared to placebo. Among the trials eligible for meta-analysis, the use of antioxidant supplements resulted in significant improvement in RQLQ scores compared to placebo [standard mean difference (SMD) -1.22; p = 0.05] and total IgE levels [SMD 0.01; p = 0.04], while there was a trend toward a reduction in antigen-specific IgE [SMD 0.18; p = 0.07] in the placebo group compared to antioxidant supplements.

CONCLUSIONS

Antioxidant supplements may be beneficial in improving symptoms and quality of life in patients with allergic rhinitis, however, current evidence remains limited due to study heterogeneity and variable outcome measures. Additional high-quality studies are needed to establish appropriate recommendations.

